# Demonstrating service delivery models for effective initiation and retention on pre-exposure prophylaxis (PrEP) among female bar workers in Dar es Salaam, Tanzania: A double randomized intervention study protocol

**DOI:** 10.1371/journal.pone.0304077

**Published:** 2024-06-27

**Authors:** Joy J. Chebet, Winfrida Onesmo Akyoo, Hannah Goymann, Guy Harling, Dale A. Barnhart, Idda H. Mosha, Doreen Donald Kamori, Monica Gandhi, Theodora Mbunda, Judith Kipeleka, David Sando, Donna Spiegelman, Rose Mpembeni, Till Bärnighausen

**Affiliations:** 1 Department of Health Promotion Sciences, Mel and Enid Zuckerman College of Public Health, The University of Arizona, Tucson, Arizona, United States of America; 2 Department of Behavioural Sciences, School of Public Health and Social Sciences, Muhimbili University of Health and Allied Sciences, Dar-es-Salaam, Tanzania; 3 Heidelberg Institute of Global Health, Medical Faculty and University Hospital, Heidelberg University, Heidelberg, Germany; 4 Institute for Global Health, University College London, London, United Kingdom; 5 Department of Epidemiology, Harvard T. H. Chan School of Public Health, Boston, Massachusetts, United States of America; 6 Africa Health Research Institute, KwaZulu-Natal, South Africa; 7 Harvard Center for Population and Development Studies, Harvard T. H. Chan School of Public Health, Cambridge, Massachusetts, United States of America; 8 MRC/Wits Rural Public Health & Health Transitions Research Unit (Agincourt), University of the Witwatersrand, Johannesburg, Gauteng, South Africa; 9 Department of Microbiology and Immunology, Muhimbili University of Health and Allied Sciences, Dar-es-Salaam, Tanzania; 10 Division of HIV, Infectious Diseases, and Global Medicine, Department of Medicine, University of California, San Francisco (UCSF), San Francisco, California, United States of America; 11 Management and Development for Health, Dar es Salaam, Tanzania; 12 Department of Global Health and Population, Harvard T.H. Chan School of Public Health, Boston, Massachusetts, United States of America; 13 Department of Biostatistics, Harvard T.H. Chan School of Public Health, Boston, Massachusetts, United States of America; 14 Department of Nutrition, Harvard T.H. Chan School of Public Health, Boston, Massachusetts, United States of America; 15 Department of Biostatistics and Center for Methods in Implementation and Prevention Science, Yale School of Public Health, New Haven, Connecticut, United States of America; 16 Department of Epidemiology and Biostatistics, School of Public Health and Social Sciences, Muhimbili University of Health and Allied Sciences, Dar-es-Salaam, Tanzania; Botswana-Harvard AIDS Research Institute, BOTSWANA

## Abstract

**Background:**

Pre-Exposure Prophylaxis (PrEP) has demonstrated efficacy in preventing HIV infection. Female Bar Workers (FBWs) often act as informal sex workers, placing them at risk of HIV infection. Despite expressing interest in PrEP, FBWs face barriers to accessing public-sector clinics where PrEP is delivered. We developed a study to compare the effectiveness of workplace-based PrEP provision to standard-of-care facility-based provision for PrEP initiation, retention and adherence among FBWs.

**Methods:**

In this double-randomized intervention study, FBWs aged 15 years and above will be screened, consented and initiated on PrEP (emtricitabine/tenofovir disoproxil), and followed for six months. Participants will be randomized at the bar level and offered PrEP at their workplace or at a health facility. Those who are initiated will be independently individually randomized to either receive or not receive an omni-channel PrEP champion intervention (support from an experienced PrEP user) to improve PrEP adherence. We expect to screen 1,205 FBWs to enroll at least 160 HIV negative women in the study. Follow-up visits will be scheduled monthly. HIV testing will be performed at baseline, month 1, 4 and 6; and TDF testing at months 2 and 6. Primary outcomes for this trial are: (1) initiation on PrEP (proportion of those offered PrEP directly observed to initiate PrEP); and (2) adherence to PrEP (detectable urine TDF drug level at 6-months post-enrollment). The primary outcomes will be analyzed using Intention-to-Treat (ITT) analyses.

**Discussion:**

Using a randomized trial design, we will evaluate two interventions aiming to reduce barriers to uptake and retention on PrEP among FBWs, a vulnerable population at risk of HIV acquisition and onward transmission. If these interventions prove effective in promoting PrEP among FBWs, they could assist in abating the HIV epidemic in Africa.

**Trial registration:**

Registered with German Clinical Trials Register (www.drks.de) on 29 April 2020; Registration number DRKS00018101.

## Background

In 2015, the World Health Organization (WHO) recommended the use of Pre-Exposure Prophylaxis (PrEP) as part of a comprehensive HIV prevention package for those at substantial risk for HIV acquisition [1]. Results from trials have shown daily oral PrEP to be highly efficacious, reducing HIV acquisition significantly when used as prescribed [2–7]. Consequently, open-label PrEP trials in high-risk populations are needed to determine feasible and acceptable methods of PrEP delivery that help promote both uptake and adherence [8]. Key questions include: (i) whether those at substantial risk are motivated and can initiate and sufficiently adhere to oral PrEP to achieve HIV prevention benefits; (ii) which delivery models are most effective; and (iii) how best to generate demand for PrEP in key populations without further stigmatizing these groups or PrEP itself.

Women who trade sex for money, goods or services–Female Sex Workers (FSWs)–are at a disproportionally greater risk of acquiring HIV due to structural, interpersonal, and behavioral risk factors, and are among key population for HIV prevention [9, 10]. While Female Bar Workers (FBWs)–women who serve food and alcohol at bars–may not self-identify as sex workers, studies indicate they often engage in intermittent transactional sex as a means to supplement their income [11–15]. These sexual encounters render FBWs vulnerable to HIV infection: (i) directly, through interaction with partners living with HIV; (ii) biologically, through frequent or recurrent Sexually Transmitted Infections (STIs), which facilitate HIV acquisition [16]; (iii) interpersonally, when FBWs cannot negotiate condom use, experience partner abuse or sexual coercion [17, 18]; and (iv) structurally, through social and political systems that promote stigma, hinder HIV prevention service provision or place women at an economic disadvantage [14, 19].

A recent pilot study interviewed and provided HIV testing and counseling (HTC) services to 66 FBWs at seven bars in Kinondoni district, Dar es Salaam municipality, Tanzania [13]. Only four of the 56 FBWs who completed HTC tested positive (7.1%). However, 35% of respondents reported engaging in sex for money with bar clients, while another 15% reported transactional sex with non-bar-based clients and 58% reported multiple non-bar-based sexual partners in the preceding 12 months. When asked, 54% of these FBWs stated being either “very interested” or “somewhat interested” in taking a daily pill to protect against HIV; this figure was 61% among those who reported having sex with a bar client for money [13].

Accessing PrEP requires frequent contact with the health system for eligibility screening, initiation and follow up for ongoing counseling and prescription refills [1]. Early studies have shown poor retention in the PrEP cascade of care, thus compromising the drug’s effectiveness [20–24]. Given the novelty of PrEP in sub-Saharan Africa, and the dearth of research on the subject, we can look to lessons from work on HIV treatment implementation science to ensure an enabling environment for PrEP retention. Notably, HIV treatment programs have moved away from facility-based treatment models to more people-centered ones that mitigate loss to follow up [25–27]. For example, the WHO endorses a differentiated service delivery model, which simplifies HIV service delivery, prioritizes the preferences of the end-user, and tailors care to meet the needs of the user [28]. ART service delivery models that decentralize HIV service provision, including community models have been shown to be efficacious, and provide a means to expand access to HIV services by overcoming barriers related to time and costs for travel to a health facility [29–31]. These models further reduce supply-side burdens by decongesting health facilities [30]. The differentiated service delivery model might be even more important for key populations and groups like FBWs who require tailored services [28].

In Tanzania, PrEP was launched in 2018 as part of the country’s fourth Health Sector HIV and AIDS Strategic Plan (HSHSP IV 2017–2022) when the government began demonstration studies to investigate the feasibility of implementing PrEP [32]. Initially, the policy specifically targeted key populations and serodiscordant couples. However, in 2019 eligibility was extended to include adolescent girls and young women at high risk of acquiring HIV [33]. Currently, PrEP provision is standard of care nationally for those at high risk of being infected by HIV due to social, economic and behavioral determinants; however, there no is effective model in Tanzanian context to be used as a standard of care to deliver PrEP to high risk FBWs. The absence of such procedure is a central reason for this study, to determine the acceptability and feasibility of PrEP provision for groups at high risk of HIV acquisition, including FBWs.

There is an urgent need to assess the feasibility of PrEP delivery for FBWs, who are at high risk of HIV acquisition and are potentially interested in PrEP. In deciding who should be offered PrEP, its benefits for HIV prevention should be balanced with possible adverse events, costs, and feasibility. In this paper, we lay out a protocol for a forthcoming double randomized implementation trial on PrEP delivery options among FBWs working in bars in Dar es Salaam, Tanzania.

## Methods

### Goal and objectives

The overall goal of this study is to evaluate the relative effectiveness of delivery modalities for initiation of, and adherence to, PrEP among FBWs in Dar es Salaam. Specifically, this trial’s central objectives are:

- Primary objective 1: To assess whether workplace-based PrEP initiation for Dar es Salaam FBWs at their bar of work increases the rate of PrEP initiation relative to initiation at HIV Care and Treatment Clinics (CTC) with the assistance of a peer navigator (PrEP champion);
- Primary objective 2: To determine whether workplace-based initiation and provision of PrEP refills to FBWs in Dar es Salaam at their bar of work increases PrEP retention and adherence relative to facility-based PrEP initiation and refilling at CTCs;
- Primary objective 3: To explore preferences for willingness, and factors influencing uptake of long-acting injectable HIV PrEP among female barmaids in Ubungo Municipality.
- Secondary objective 1: To assess whether receiving support by omni-channel (physical and virtual) PrEP champion, improves PrEP adherence relative to the national standard of care.
- Secondary objective 2: To evaluate the performance of a novel point of care urine assay as a measure of adherence to PrEP.

### Study site and health facility selection

**Fig 1:**
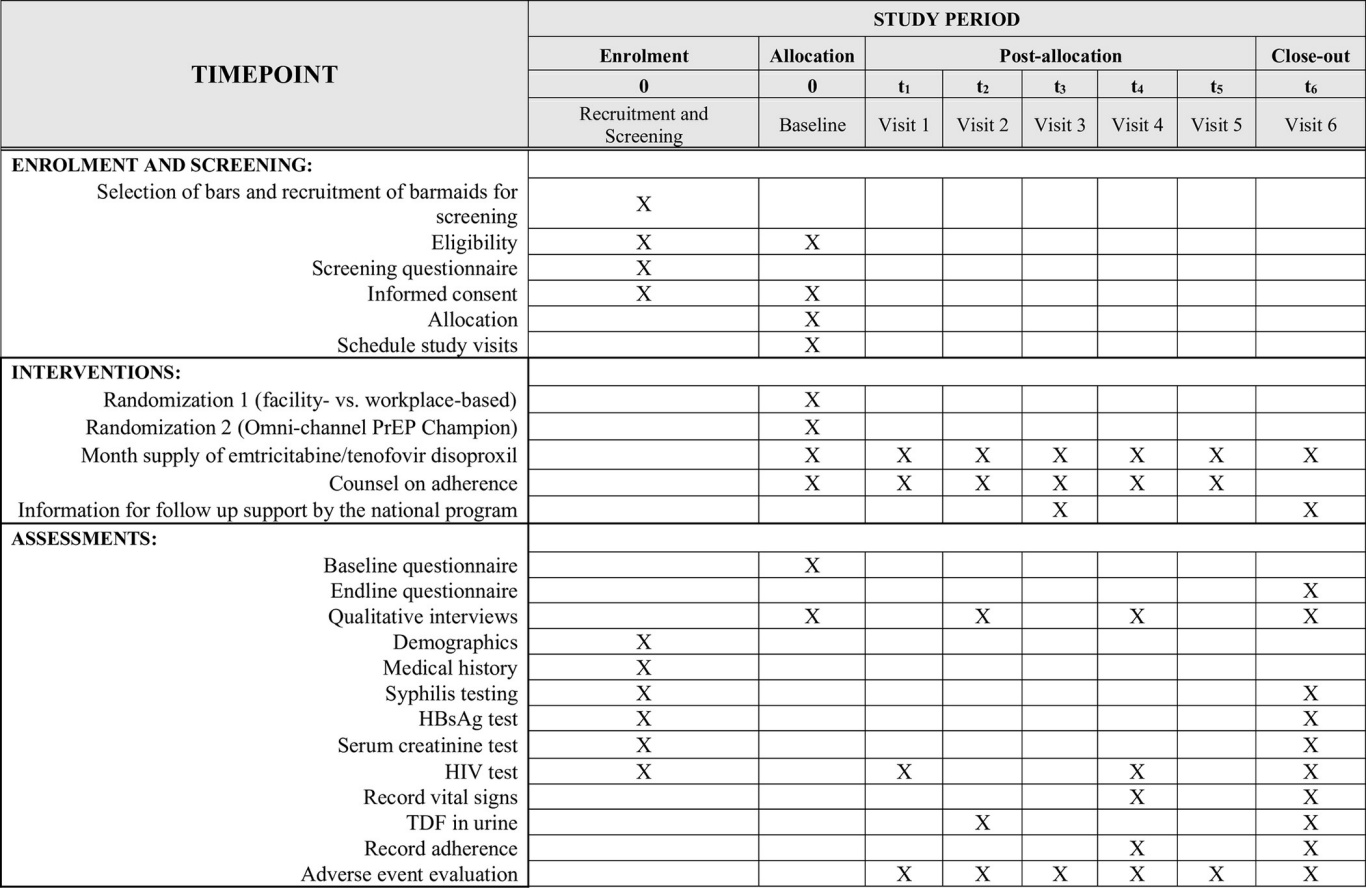
Study schedule of enrollment, interventions and assessments.

This study will be conducted in the Ubungo Municipality of Dar es Salaam region. Mapping of all registered bars will be done to get a list of all bars in the district. This list will be used as a sampling frame and simple random sampling technique will be used to select 146 study bars. The bars selected for the study will be randomly allocated into workplace or facility arm at a ratio of 2:5 (Fig 1). Four health facilities will be selected to participate in this trial. Facilities selected will have: (i) existing HIV care and treatment services; (ii) a well-established antiretroviral (ARV) drug supply chain; (iii) health care providers trained on PrEP provision; and (iv) existing HIV prevention services, including condom distribution and comprehensive Positive Health Dignity Prevention (PHDP) services [34]. At each selected facility, we will identify two PrEP providers; one HIV nurse counselor [registered nurses] and one clinician [clinical officer] trained on the use of ARVs for treating and preventing HIV infection as per WHO consolidated guidelines and implementation framework for PrEP in mainland Tanzania [28, 35]. These staff will be paid for the overtime work they will undertake in supporting this study. The clinicians will be responsible for taking a clinical history and prescribing PrEP to all study participants. The nurse counselors will be responsible for providing adherence counseling and conducting client follow-up for PrEP refills. To ensure normal functioning of health facilities, study activities will take place after standard clinic hours, and in periods of low facility utilization.

### Recruitment criteria and screening

Participant recruitment will be carried out by visiting the licensed bars selected. After an initial telephone conversation with the license holder or representative, the study team–who have a graduate-level academic background, and who will have been trained in ethics, quantitative and qualitative methods–will arrive at the bar and work with the on-duty manager (discreetly and in private) to make a list of all women who work as FBWs serving drinks directly to customers. The bar manager will be provided 10,000 TZS (~3 USD) as compensation for the time taken to complete the listing and for the inconvenience of the study team’s presence. Study staff will emphasize the compensation will be provided regardless of the bar or barmaid participation in the study. Every woman on the developed list will then be invited to participate in a screening process. This includes undergoing an informed consenting process, where study staff will discuss that participation in the study is entirely their choice and that they are free to withdraw from the study at any time.

Consenting barmaids will complete a questionnaire and undergo HIV testing and counseling (HTC) (Fig 1). The risk of HIV acquisition will be assessed using a national eligibility screening questionnaire, developed as part of the National Framework for PrEP [35]. This screening tool considers having multiple partners, sex work, and condomless sex with a non-permanent partner as increasing the risk of HIV infection. FBWs will be interviewed within private mobile vehicles with tinted windows or in a private location proposed by the FBWs or on-duty manager to maintain confidentiality. At the initial recruitment stage, potential participants will be offered 10,000 TZS (~3 USD) to compensate them for the time spent away from their work while completing screening activities. This will be provided at the end of the screening process, unconditional on participation in the randomized study. Permission and support for the study will be obtained from the Ubungo Municipal medical officer.

Participants will be excluded from the study based on positive HIV status, medical conditions that may be exacerbated by PrEP use, and any use of contraindicating medication. Inclusion/exclusion criteria used to determine participant eligibility for the trial are listed in Table 1.

**Table 1:** Summary of baseline trial inclusion and exclusion criteria.

Inclusion Criteria	Exclusion Criteria
• Identify as female and report being aged 15 years or older; and • Working in a licensed bar in Ubungo municipal council as a barmaid or server; and • Willing to comply with all study procedures and remain within Dar es Salaam for the duration of the study (6 months); and • Able to take oral medication and willing to adhere to the medication regime; and • Provide signed and dated written informed consent or a thumb print if unable to read/write; and • Eligible for PrEP as per essential criteria for Adolescent girls and young women outlined in the Implementation Framework for Pre-Exposure Prophylaxis of HIV in Tanzania Mainland (2021); and • Have working experience of at least 2 months.	• Test seropositive for HIV or refuse HIV counseling and testing; or • Report past diagnosis of renal disease, diabetes, or hypertension; or • Report flu-like symptoms that may represent acute HIV infection; or • Report currently taking any of the following medications: any medicine containing emtricitabine or tenofovir disoproxil fumarate; any medicine containing tenofovir alafenamide; any medicine containing lamivudine; nephrotoxic medication; adefovir; didanosine; atazanavir; ledipasvir with sofobuvir; darunavir; or lopanavir with ritonavir; or • Report bone pain or other bone problems; or • Have abnormal renal function (serum creatinine <60ml/min); or • Test positive for Hepatitis B virus infection based on a hepatitis B surface antigen (HBsAg) test; or

### Study procedures

To assess participant eligibility, a series of clinical tests will be performed (Fig 1). These include a finger-prick blood sample for point-of-care HIV and Hepatitis B Surface Antigen (HBsAg) testing to determine chronic hepatitis B infection; and a blood draw for laboratory-based serum creatinine and syphilis STIs testing. The test will be administered during screening and again at the end of the study, to serve as a proxy for condom less sex and a signifier of risk compensation, defined as amplified risk taking as a result of perceived protection following adoption of PrEP [36, 37]. The results of all rapid tests will be provided to study participants at the time of testing. Serum creatinine test results will be discussed with participants if the results are abnormal. Baseline creatinine test will be analyzed at the Muhimbili University of Health and Allied Sciences (MUHAS) laboratory, which is accredited as adhering to Good Clinical Laboratory Practice (GCLP) standards. Additionally, a medical history of each participant–including current use of any over-the-counter medications–will be taken by study personnel. Based on this, the study personnel will assess if the prospective participant is taking any PrEP-contraindicated medications as per WHO recommendations. To gain a sense of general health, a brief physical examination for vital signs, including height, weight and blood pressure will be completed by a clinical team at a private location on or near the study bar premises, or a study health facility (depending on the treatment group).

Standard HIV counseling will be provided to participants whether they test positive or negative, per national Tanzanian guidelines [34]. Standard HTC includes pre-test counseling, a finger-prick based rapid HIV test, followed by a second confirmatory test Uni-Gold should the first test prove positive and post-test counseling. Individuals who test positive for HIV will be linked to HIV care and treatment services at a local HIV CTC. All who test negative for HIV and are eligible as per the National PrEP Framework, will be offered the opportunity to participate in this trial [35].

PrEP, formulated as tenofovir disoproxil fumarate 300 mg and emtricitabine 200 mg, will be prescribed to consenting participants meeting eligibility criteria, to be taken orally as a pill once per day. This will be observed the first time the pill is taken at initiation. At each visit, participants will receive counseling (including information on adherence) by a study PrEP provider (registered nurse or clinical officer). Adherence to emtricitabine/tenofovir disoproxil will be measured in three ways. First will be by self-reported adherence in terms of percentage of doses taken at the agreed time of day and on the correct day. This will be assessed by study staff using questions in the midline and endline questionnaire, asking ‘in the last 30 days, on how many days did you miss a dose of your PrEP medication? how long ago was your last missed dose?’. Second will be by pill count, where study PrEP providers will record the number of returned pills at the visit. Third will be by a point-of-care urine assay test for TDF, administered at month 2 and 6.

A novel point-of-care urine assay will be used as a measure of adherence to PrEP in this trial at 2 and 6 months follow up (Fig 1). Current tests for adherence to TDF–an active agent in PrEP–are predominantly blood (plasma/antibodies) or hair based [38, 39]. These traditional methods are invasive and/or require specialized personnel, equipment and laboratory facilities. The University of California San Francisco (UCSF), in collaboration with Alere Rapid Diagnostics, has developed a low-cost rapid urine assay that does not require specialized equipment or training. This urine-based assay is 96% sensitive, 100% specific and precise (<15% coefficient of variation) in previous studies [38, 39]. The test is based on the chromatographic immunoassay principle. The urine rapid test for adherence measures the presence or absence of adequate TDF in the urine whereby a positive test result indicates the presence of adequate TDF concentration (˃1500ng/ml) in the urine implying good adherence to PrEP while a negative test result indicates the absence of adequate TDF concentration in the urine (<1500ng/ml) implying ‘poor-adherence to PrEP. The present trial will utilize this point-of-care urine assay and evaluate its performance in a real-world setting.

Patient-reported outcomes, including PrEP adherence and use, will be ascertained through a short questionnaire at each trial visit, in addition to the collection of baseline data prior to enrollment (Fig 1). Quantitative surveys will be conducted at the baseline (month 0), midline (month 2) and endline (month 6). At baseline, a post consent survey will be conducted with all participants meeting the eligibility criteria and will capture demographic, socioeconomic, work and sexual/reproductive health information. At enrollment, a survey will be conducted among eligible participants to assess their motivation to accept or decline the use of PrEP and enrollment in the study. At midline and endline study periods, additional surveys will be administered, to inquire about changes to social and sexual practices since initiating PrEP, and any side effects experienced. According to the national guidelines, participants will receive PrEP refills once per month for the first six months then depending on their adherence they are given a three-month refill.

### Participant withdrawal and termination

Participants will be free to withdraw from participation in the study at any time at their own request. This will be explained clearly during the consent process.

An investigator will terminate participation in the study and stop provision of PrEP if:

- A participant tests positive for HIV (the participant will be offered counseling and linked to HIV CTC services);
- A participant’s renal function drops abnormally low, below <60ml/min;
- A participant experiences any clinical adverse event(s) (AE) or laboratory abnormality;
- A participant ceases to work as a FBW in study bar.

### Qualitative interviews

Semi-structured, in-depth interviews (IDI) will be conducted with a subset of FBWs (n = 30) enrolled in the larger trial at baseline, midline and end line to contextualize clinically derived adherence results (Fig 1). These interviews will explore understandings of adherence, reasons for non-adherence, challenges to adherence, and strategies and recommendations to improve adherence. Additional questions will explore the FBW’s understanding of PrEP, the drug’s mechanism of action, perceived drug efficacy against HIV acquisition, and if this perception (positive or negative) influences their adherence to PrEP. Also, barmaids who have been successfully using PrEP pills for six months and those who have not been involved with PrEP intervention will be invited for interviews on their preferences, willingness to use and factors that may influence uptake of long-acting injectable PrEP.

Drawing on the FBW’s personal experience and those of their friends, interviews will also explore trends in PrEP use (drug self-regulation), changes in perceived risk, and general attitudes towards the drug. During the endline survey, we will also collect information from barmaids, PrEP champions and health care workers regarding perceptions, personal understanding and experience on the omni-channel PrEP champions intervention. Key informant interviews will be conducted with stakeholders like NGOs, health care providers, National AIDS Control Program who are working in HIV interventions to learn their views on preferences, willingness to provide and feasibility for implementation of long-acting injectable PrEP.

We will use a purposive sampling design for qualitative interviews, selecting women to be invited to participate to promote diverse perspectives and experiences. We will invite women of varying ages, length of employment, marital status, wealth, sexual behavior and other factors established during preliminary fieldwork. We will recruit women in part based on sexual practices disclosed in the baseline questionnaire putting them at greater risk of HIV acquisition, specifically: having multiple sexual partners; receiving payment for sex in the form of money, goods or services; and low condom use. Health care workers and other key stakeholders working in HIV will be selected using communication from the municipal social welfare officer and District HIV Coordinator who will help to provide information on stakeholders working in HIV. To maintain participant confidentiality and ensure data integrity, follow-up interviews will be conducted in a hired car or a private and silent room in, or close to the bar.

### Sample size and randomization

This study will be powered to detect differences of a clinically significant magnitude with 80% power in: (1) initiation; and (2) adherence across the workplace-based and facility-based PrEP provision groups. Sample size calculations were performed in Stata 15 (StataCorp, College Station TX). We used a two-sided Type-I error of *α* = 0.05 for all calculations.

**Fig 2:**
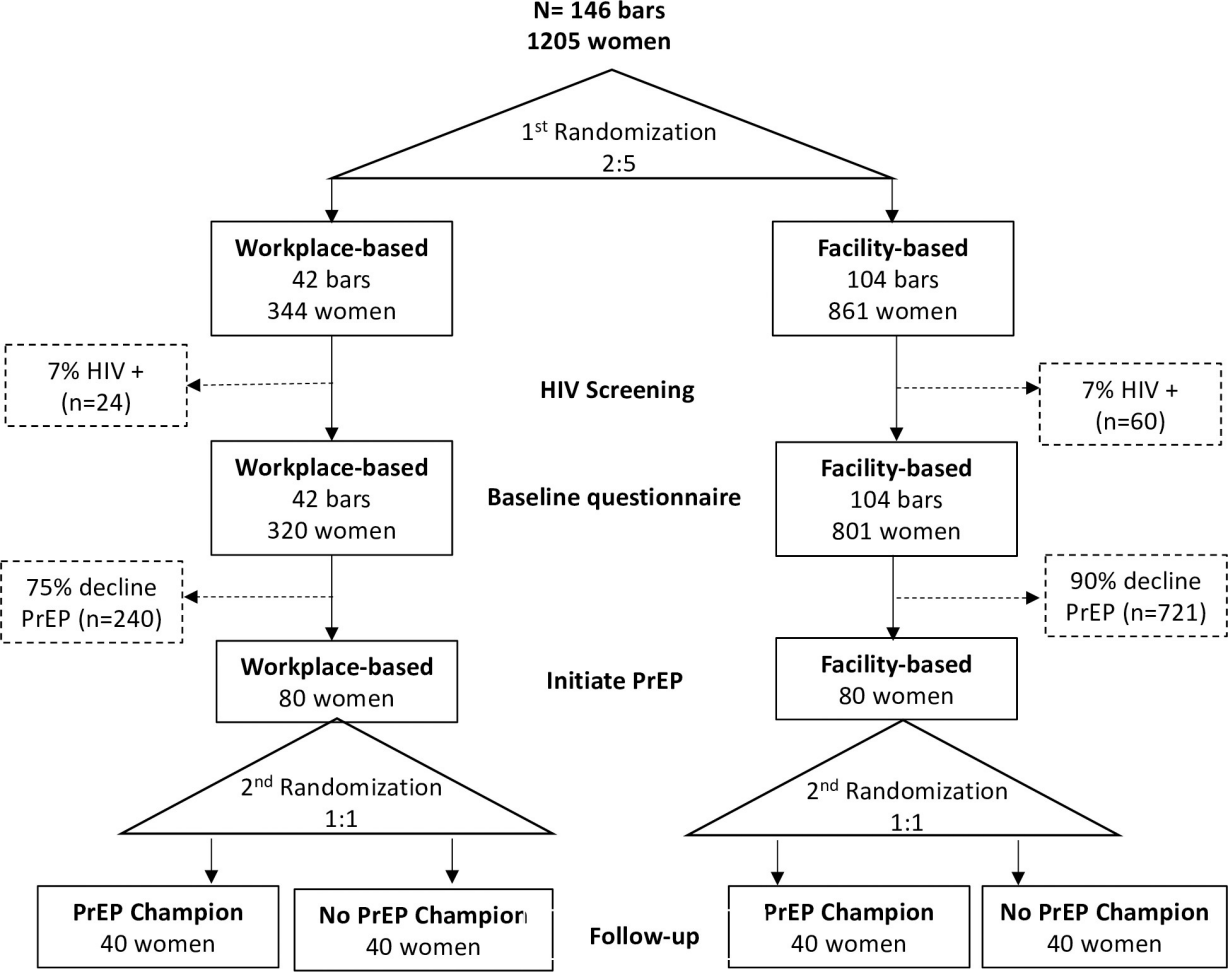
Randomization and group assignments.

The study will involve two sequential, independent randomizations. First, at the time of screening, bars will be randomized into either workplace-based or facility-based PrEP initiation and follow-up. Second, participants will be concurrently individually randomized into either receiving support from an omni-channel PrEP champion or not, for adherence and follow-up support. For initiation, the outcome will be the percentage of HIV-negative women screened who are directly observed to initiate PrEP. Our null hypothesis is that there is no difference in initiation rates across study arms, and our alternate hypothesis is that the initiation rates differ in the two study arms. Based on data from our pilot study showing that 34% of the responding FBWs reported being “very interested” in oral PrEP and a further 20% “somewhat interested”, we assume that PrEP initiation will be approximately 25% in the workplace-based arm and 10% in the facility-based arm. To obtain sufficient power for our second primary outcome of PrEP adherence, we will randomize 146 bars in a 2:5 ratio to the facility-based arm and workplace-based arms respectively (Fig 2). Assuming, based on our pilot data the average bar employs 8.25 FBWs and that 7% of screened women are HIV-positive, we anticipate screening approximately 1205 women and identifying 1120 women who are HIV-negative and eligible for PrEP.

**Fig 3:**
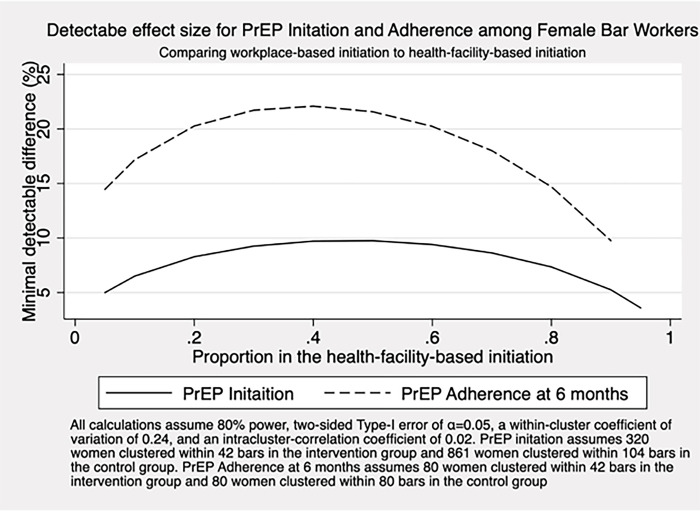
Minimum detectable effect size for PrEP initiation and adherence among Female Bar Workers, comparing workplace-based initiation to facility-based initiation.

Assuming 320 women clustered within 42 bars in the intervention group, 861 women clustered within 104 bars in the facility-based group, the size of the coefficient of variation between clusters of 0.24 as observed in our pilot study [40], and an Intracluster-Correlation Coefficient (ICC) of 0.02, we would anticipate having at least 80% power to see a 10 percentage point difference in initiation rates over a wide range of PrEP initiation rates in the control group (Fig 3). An ICC of 0.02 reflects the ICC for using modern family planning methods excluding condoms in our pilot study and also corresponds with previous research on the ICC of biomarkers in Dar es Salaam [41]. However, the number of participants required to achieve 80% power is not sensitive to the ICC. For adherence, we will compare proportions of who are adherent to PrEP between the facility-based vs. workplace-based arms at 6 months following initiation. Participants lost to follow-up before 6 months will be assumed to be non-adherent. Accounting for clustering as above and based on our randomization ratio and the anticipated enrollment rates for each arm (Fig 3), we anticipate enrolling 160 women, 80 in each arm, which will give us at least 80% power to detect a difference of 23 percentage points between arms. Should there be fewer than 1120 women eligible in the original 146 bars, we will expand the number of bars to include neighboring districts–continuing to randomize them in the ratio 2:5 –until the required number of eligible women is reached.

### Blinding

The study cannot be blinded. Participants will know which of the four study groups they are in based on the intervention they receive. Investigators will know who is receiving and not receiving workplace-based PrEP delivery and PrEP champion support. However, the data collected will be de-identified and study arms as well as bar names will be blinded during interim analysis.

### Outcome measures

Study primary and secondary trial endpoints will be assessed using the following measures:

1. Initiation on PrEP: measured as the proportion of those offered PrEP who are directly observed to initiate PrEP. This endpoint will assess whether the workplace-based initiation process improves uptake, and thus improves the ability of PrEP programs to protect those at high risk of HIV acquisition.
2. Adherence to PrEP: measured as urinary TDF detectability (Yes/No) at 2- and 6-months post-enrollment. The primary endpoint will be measured in the two delivery method groups: facility-based and workplace-based PrEP delivery. Women who default from PrEP treatment or are lost to follow up will be assumed to have indetectable urinary TDF. Loss to follow up will be defined as a participant who is no longer working in the bar, in Ubungo municipality and not reachable by any means.
3. Retention in PrEP care: measured as refill rates at each study visit as a process outcome related to the primary objective. This secondary endpoint will be analyzed in two ways: (i) comparing the two PrEP champion groups (participation and non-participation) to evaluate secondary objective 2; and (ii) comparing facility- and workplace-based PrEP delivery groups. Retention in care reflects the proportion of those initiating care who remain accessible to programs, and thus potentially able to take PrEP. Previously, without support from a peer navigator, ART linkage rates in Dar es Salaam public-sector implementation projects have been as low as 30%. Peer navigators referred to as champions have been used by various implementing partners including Management and Development for Health in case finding and tracking in HIV interventions. These will be involved for identification, active linkage, counseling and follow up of the barmaids. PrEP champions will be responsible for close follow up by creating a good rapport and keeping close communication to assigned group of champions.
4. Performance of a novel point of care urine assay: The point-of-care urine test is a rapid chromatographic immunoassay based on the principle of competitive binding. The quality control of this test will be ensured as follows. First, the test has an inbuilt quality control component that will be observed during each test. Quality control will also be assured through external quality control, by comparing positive control from a known PrEP client with good TDF adherence and negative control (participants with no history of taking PrEP). For this test project staff (clinical officers, laboratory technicians and registered nurses) will be trained on the procedures for testing and quality control.

### Data management

Clinical and quantitative data will be collected using encrypted tablet computers through the digital data collection application, Open Data Kit (ODK) [42]. The data, automatically uploaded to the application cloud server, will be encrypted and password protected. Data will be uploaded nightly to the application server using Wi-Fi. Quality Control (QC) procedures will be implemented beginning with the data entry system and data QC checks that will be run on the database will be generated and reviewed weekly by remote study staff. Any missing data or data anomalies will be communicated to the study site for clarification and resolution. Qualitative interviews will be audio recorded and de-identified data saved to a secure, study-specific, password-protected computer. All paper-based trial material will be stored securely in the study offices under lock and key.

### Data analysis

#### Statistical analyses

Primary endpoint 1, PrEP initiation will use an Intention-to-Treat (ITT) analysis, based on all participants who were eligible to enroll in the trial and consented. We hypothesize that the proportion of eligible individuals who agree to initiate PrEP and are observed to take the first tablet on day 0 will be higher in the workplace-based group than in the facility-based group. The null hypothesis is of no difference in directly observed initiation rates between the workplace-based and facility-based group. Generalized estimating equations for binary data accounting for clustering within bars, using a compound symmetry working correlation matrix with a log link, will be used to assess this endpoint.

Primary endpoint 2, PrEP adherence will also use ITT analysis, among participants who initiated PrEP. For Primary endpoint 2, we hypothesize that the urinary TDF detectability in those enrolled in the workplace-based study group will be significantly higher than that in the facility-based group when measured at the 6-month post-initiation visit. The null hypothesis is of no difference between the workplace- and facility-based groups. We will assume that women who default from treatment or are lost to follow-up will have indetectable TDF, since accessing medication outside of this study is currently unlikely in Tanzania. Generalized estimating equations for binary data accounting for clustering within bars, using a compound symmetry working correlation matrix with a log link will be used to assess this endpoint.

The number of refills per initiated participant (secondary endpoint 1) will be measured as 0, 1, 2, 3, 4, 5, 6 denoting the number of return visits at which emtricitabine/tenofovir disoproxil was dispensed to the participant. The endpoint will be assessed in a single generalized estimating equation for continuous data accounting for clustering within bars. A compound symmetry working correlation matrix with an identity link and Poisson distribution will be used to assess this endpoint. The models will include a term for location of PrEP delivery (workplace or facility) and PrEP champion support (yes/no). In secondary analysis, the interaction between the two interventions will be assessed by introducing a cross-product term between the two intervention components, and its significance will be evaluated by a robust core test Bonferroni corrected vs. detectable.

To assess the performance of a novel point of care urine assay as a measure of adherence to PrEP (secondary endpoint 2), the interpretation of the test onsite will be compared with those conducted by the test’s designers as the gold standard. The extent to which the two align will be compared using spearman and Pearson correlations for the measured values, and an odds ratio and Reacma test for the binary detectable/not detectable variable.

#### Qualitative analyses

An inductive approach, founded on grounded theory, will underpin the qualitative data collection and analysis process [43]. A list of general questions on the participant’s experience with PrEP will be used to guide IDIs. These questions will be pilot tested and adapted to ensure their understandability and applicability. Following each day of interviews, research assistants and investigators will convene for a post-interview debriefing session. During this time, emerging themes, areas of further exploration and saturated topics will be identified [44]. Following data collection, audio recordings will be transcribed verbatim and translated from *Kiswahili* to English by hired experienced transcribers then data collectors will review for correctness. The qualitative researcher in the study will review the transcripts for quality.

All IDIs will be quality controlled by bilingual researchers to ensure that the content of the recorded interviews is reflected in the transcripts. This will aide in triangulation of findings and provided texture and nuance to descriptions. To maintain the context and meaning of resulting data, transcripts will be thematically analyzed in their original language by bilingual researchers. A list of codes, founded on the trial’s objectives, will be developed from a subset of rich and representative interviews. These codes will be applied to all transcripts. Data will be coded and analyzed using NVivo version 14. During the analysis process, a subset of co-authors will discuss codes and themes, and draw comparisons across respondent groups and where they receive PrEP services. Throughout this process, research assistants and study investigators will hold consensus and analysis workshops to ensure data interpretation is reflective of participant sentiment. Data will then be summarized by theme for reporting.

### Ethics and trial registration

This study has received ethical approval from the Tanzanian National Institute of Medical Research (NIMR/HQ/R.8a/Vol IX/3348), MUHAS (DA.282/298/01.C/) and Heidelberg University (S-687/2019). The trial has been registered with the German clinical trials register (DRKS00018101). All participants will provide written informed consent prior to enrollment. This will involve a verbal explanation, in terms suited to potential participants’ comprehension, of the purposes, procedures and potential risks of the study and of their rights as research participants. Participants will have the opportunity to carefully review the written consent form and ask questions prior to signing. Researchers will emphasize the optional nature of participation and inform participants that they are able to withdraw from the study at any time. Only consenting FBWs will be enrolled in the study. Assent for minors was waived for this study since female bar workers aged 15 years and above in Tanzania are considered emancipated minors. Additionally, the are prioritized for HIV prevention services as they fall within a vulnerable population (Adolescent Girls and Young Women) as indicated in the national guidelines for the management of HIV and AIDS [34, 35]. For non-literate participants, a thumbprint, provided in the presence of a literate witness of their choosing, will be accepted.

Safety oversight will be under the direction of a Data Safety and Monitoring Board (DSMB), composed of individuals with the appropriate expertise, including a Tanzanian clinician, clinicians with experience working on antiretroviral treatment or prevention projects and a biostatistician. The DSMB will meet prior to the start of enrollment and at midline, to assess safety and efficacy data on each arm of the study. The DMSB will operate under the rules of an approved charter that will be written and reviewed at the organizational meeting of the DSMB. At this time, each data element that the DSMB will assess will be clearly defined. The study statistician (GH), and other study personnel under his supervision, will work with the DSMB to produce reports and interim analyses as requested.

### Trial status and dissemination plan

Enrollment for the present trial is anticipated to commence in the summer of 2023, wherein a three-month rolling enrollment period will begin. Participants meeting eligibility will be followed for six months, with the trial anticipated end date of 6 months following the last enrollment. Study investigators are committed to sharing findings from this study locally in Tanzania with the National AIDS Control Program, National Institute for Medical Research and other relevant governmental bodies developing national PrEP guidelines. Results will also be shared more broadly to global policy and research stakeholders through formal reports, academic publications, and oral presentations.

## Discussion

PrEP, a efficacious biomedical HIV prevention intervention, should be able to contribute to reducing the global HIV burden, especially among those at highest risk [20]. However, low uptake and adherence will stop PrEP’s full potential from being realized [21–24]. FBWs are not currently identified as a key population for HIV prevention by WHO, but they are at substantial risk of HIV acquisition and transmission due to multiple partners in their personal lives and engagement in transactional sex with bar patrons and others [13–15]. Dar es Salaam FBWs’ interest in a pill that prevents HIV acquisition suggests that PrEP may be acceptable in this setting. This study will use a randomized trial design to investigate whether workplace provision and text message follow-up reminders can increase PrEP initiation and adherence among Dar es Salaam FBWs. Our study will help evaluate the viability of PrEP delivery mechanisms; how delivery affects initiation and adherence; and the performance of a novel urine-based test as a measure of PrEP adherence.

FBWs typically remain in their position for a short duration of time, engaging in transactional sex until they are able to attain better job prospects [13, 15]. PrEP is therefore an ideal, user-led preventive intervention that can be used during this period of risk and then discontinued. This study will provide evidence as to whether barriers to initiation and retention can be lowered sufficiently that FBWs can use PrEP throughout this transient risk period.

One barrier to effective PrEP protection is suboptimal use. Rapidly adherence measurement is thus vital to ensuring appropriate counseling and assistance can be provided during healthcare visits. This study will provide evidence as to the feasibility of using a urine-based point-of-care TDF test. As PrEP is adopted more widely, user-friendly and accurate adherence measures will be needed [39]. Findings from our study will contribute to this evidence base.

Maximizing opportunities for uptake of PrEP worldwide for those at risk of HIV acquisition will require innovative and locally appropriate delivery methods–taking account of local HIV epidemiology, user profile, capacity, and available resources [45, 46]. Should this study demonstrate that workplace-based PrEP delivery models can be effective, wider use of such methods could be considered–both in bars and in other workplaces where staff or clientele are at high risk of HIV acquisition of transmission.

More broadly, this work will have implications and shed light on ways to adapt people-centered models shown to be effective for ART to PrEP programs [28]. As Tanzania and much of sub-Saharan Africa undertakes demonstration and feasibility studies on widespread use of PrEP, this study will contribute a client-focused model of PrEP service delivery to the policy discourse, including tailoring services to clientele, and augment existing facility-based service provision. All this should help maximize the benefits of PrEP to populations most affected by HIV.

## Supporting information

S1 Checklist SPIRIT 2013 checklist: Recommended items to address in a clinical trial protocol and related documents*. (DOC)

S1 File (PDF)

## List of abbreviations

AE: Adverse Event
ARV: : Antiretroviral
CTC: Care and Treatment Clinic
DSMB: Data Safety and Monitoring Board
FBW: Female Bar Worker
FSW: Female Sex Worker
GCLP: Good Clinical Laboratory Practice
HBsAg: Hepatitis B surface Antigen
HTC: HIV Testing and Counseling
ICC: Intracluster-Correlation Coefficient
IDI: In-Depth Interview
ITT: Intention To Treat
MUHAS: Muhimbili University of Health and Allied Sciences
NIMR: National Institute for Medical Research (Tanzania)
PHDP: : Positive Health Dignity Prevention
SMS: Short Message System
STI: Sexually Transmitted Infections
TDF: Tenofovir Disoproxil Fumarate
QC: Quality Control
UCSF: University of California San Francisco
