# Supplementary material for: Demonstrating service delivery models for effective initiation and retention on pre-exposure prophylaxis (PrEP) among female bar workers in Dar es Salaam, Tanzania: A double randomized intervention study protocol
Source: PLoS One. 2024 Jun 27;19(6):e0304077. doi: 10.1371/journal.pone.0304077 (PMC11210872; doi:10.1371/journal.pone.0304077)
Supplement: S1 File — (PDF) [file pone.0304077.s002.pdf]

Protocol Title:

DEMONSTRATING SERVICE DELIVERY MODELS FOR EFFECTIVE INITIATION AND RETENTION ON PRE-EXPOSURE PROPHYLAXIS AMONG BARMAIDS IN DAR ES SALAAM

Version	4.0
Version date	02 December 2022
Research team	
Principal Investigators	- Prof. Till Bärnighausen, MD, ScD,till.baernighausen@uni-heidelberg.de<br>- Prof. Rose Mpembeni, PhD,rcmpembeni@gmail.com<br>- Prof. Donna Spiegelman, ScD,donna.spiegelman@yale.edu<br>- Dr. David Sando MD, MSc, ScD, dsando@mdh.or.tz
Co-investigators	- Dr. Guy Harling, MA, MPH, ScD,g.harling@ucl.ac.uk<br>- Dr. Monica Gandhi, MD, MPH, Monica.Gandhi@ucsf.edu<br>- Dr. Doreen Kamori, PhD,doreenkamori@gmail.com<br>- Dr. Idda Mosha, PhD, ihmosha@yahoo.co.uk<br>- Dr. Dale Barnhart, ScD,dab000@mail.harvard.edu<br>- Dr. Theodora Mbunda, MD, PhD, tmunda@mdh.or.tz<br>- Dr. Joy Chebet, DrPH,jchebet@email.arizona.edu<br>- Prof. Albrecht Jahn, MD, PhD, MSc, *Albrecht.Jahn@uni-heidelberg.de*<br>- Dr. Sandra Barteit, MSc, MA, barteit@uni-heidelberg.de<br>- Ms. Winfrida Onesmo, MSc BC, akybless@gmail.com<br>- Dr. Judith Kipeleka, MD, jkipelekamdh.or.tz<br>- Ms. Hannah Goymann, hannah.goymann@uni-heidelberg.de
Responsible biostatistician	Dr. Guy Harling
Funding	National Institutes of Health (NIH)

16.12.22

Date and Signature of the principal investigator (Prof. Till Bärnighausen)

Table of Contents

LIST OF ABBREVIATIONS 6

1. PROTOCOL ABSTRACT 7

2. INTRODUCTION: BACKGROUND INFORMATION AND LITERATURE REVIEW 8

2.1 BACKGROUND INFORMATION 8
2.2 PROBLEM STATEMENT 10
2.3 RATIONALE 10
2.4 POTENTIAL RISKS AND BENEFITS 11
2.4.1 Known Potential Risks 11
2.4.2 Known Potential Benefits 12

3. STUDY OBJECTIVES 12

4. METHODOLOGY 14

4.1 DESCRIPTION OF THE STUDY DESIGN 14
4.2 QUANTITATIVE SURVEYS AND QUALITATIVE INTERVIEWS 18
4.3 SCHEMATIC OF STUDY DESIGN 20
4.3.1 Primary Endpoint 21
4.3.2 Secondary Endpoints 21
4.3.3 Exploratory Endpoints 22

5. STUDY ENROLLMENT AND WITHDRAWAL 22

5.1 PARTICIPANT INCLUSION CRITERIA 22
5.2 PARTICIPANT EXCLUSION CRITERIA 23
5.3 STRATEGIES FOR RECRUITMENT AND RETENTION 24
5.4 PARTICIPANT WITHDRAWAL OR TERMINATION 24
5.4.1 Reasons for Withdrawal or Termination 24
5.4.2 Handling of Participant Withdrawals or termination 25
5.5 PREMATURE TERMINATION OR SUSPENSION OF STUDY 25

6. STUDY AGENT 26

6.1 STUDY AGENT(S)AND CONTROL DESCRIPTION 26
6.1.1 Acquisition 26
6.1.2 Formulation, Appearance, Packaging, and Labeling 26
6.1.3 Product Storage and Stability 26
6.1.4 Preparation 26
6.1.5 Dosing and Administration 27
6.1.6 Route of Administration 27
6.1.7 Starting Dose and Dose Escalation Schedule 27
6.1.8 Dose Adjustments/Modifications/Delays 27
6.1.9 Duration of Therapy 27
6.1.10 Tracking of Dose 27

7. STUDY PROCEDURES AND SCHEDULE 27

7.1 STUDY PROCEDURES/EVALUATIONS 27
7.1.1 Study specific procedures 27

7.1.2 Standard of care study procedures ... 28
7.2 Laboratory Procedures/Evaluations ... 28
7.3. Study Schedule ... 29
7.3.1 Screening ... 29
7.3.2 Enrollment/Baseline ... 30
7.3.3 Follow-up ... 30
7.3.4 Final Study Visit ... 31
7.3.5 Early Termination Visit ... 32
7.3.6 Schedule of Events Table ... 32
7.4 Concomitant Medications, Treatments, and Procedures ... 33
7.5 Prohibited Medications, Treatments, and Procedures ... 33
7.6 Prophylactic Medications, Treatments, and Procedures ... 33
7.7 Rescue Medications, Treatments, and Procedures ... 33
7.8 Participant Access to Study Agent At Study Closure ... 33
8. ASSESSMENT OF SAFETY ... 34
8.1 Specification of Safety Parameters ... 34
8.1.1 Definition of Adverse Events (AE) ... 34
8.1.2 Definition of Serious Adverse Events (SAE) ... 34
8.1.3 Definition of Unanticipated Problems (UP) ... 34
8.2 Classification of an Adverse Event ... 35
8.2.1 Severity of Event ... 35
8.2.2 Relationship to Study Agent ... 35
8.2.3 Expectedness ... 36
8.3 Time Period and Frequency for Event Assessment and Follow-Up ... 36
8.4 Reporting Procedures ... 36
8.4.1 Adverse Event Reporting ... 36
8.4.2 Serious Adverse Event Reporting ... 37
8.4.3 Unanticipated Problem Reporting ... 37
8.4.4 Events of Special Interest ... 38
8.5 Study Halting Rules ... 38
8.6 Safety Oversight ... 38
9. STATISTICAL CONSIDERATIONS ... 38
9.1 Statistical and Analytical Plans ... 38
9.2 Statistical Hypotheses ... 39
9.3 Sample Size ... 39
9.4 Measures to Minimize Bias ... 42
9.4.1 Enrollment/ Randomization/ Masking Procedures ... 42
9.4.2 Evaluation of Success of Blinding ... 43
9.4.3 Breaking the Study Blind/Participant Code ... 43
10. DATA HANDLING AND RECORD KEEPING ... 43
10.1 Data Collection and Management Responsibilities ... 43
10.2 Study Records Retention ... 44
10.3 Protocol Deviations ... 44
10.4 QUALITY ASSURANCE AND QUALITY CONTROL ... 44
10.5 Analysis Datasets ... 45
10.5.1 Description of Statistical Methods: General Approach ... 45
10.5.2 Analysis of the Primary Efficacy Endpoint(s) ... 45

10.5.3 Analysis of the Secondary Endpoint(s) 46
10.5.4 Adherence and Retention Analyses 46
10.5.5 Baseline Descriptive Statistics 46
10.5.6 Additional Sub-Group Analyses 47
10.5.7 Tabulation of Individual Response Data 47

11. ETHICS/PROTECTION OF HUMAN SUBJECTS 47

11.1 ETHICAL STANDARD 47
11.2 DECLARATION OF HELSINKI 48
11.3 INSTITUTIONAL REVIEW BOARD 48
11.4 INFORMED CONSENT PROCESS 48
11.4.1 Information Documents Provided to Participants 48
11.4.2 Consent Procedures and Documentation 48
11.5 PARTICIPANT AND DATA CONFIDENTIALITY 50
11.5.1 Research Use of Stored Human Samples,Specimens or Data 50
11.6 FUTURE USE OF STORED SPECIMENS 51

12. PUBLICATION AND DATA SHARING POLICY 51

13. STUDY ADMINISTRATION 51

13.1 STUDY LEADERSHIP 51

14. CONFLICT OF INTEREST POLICY 51

15. BUDGET AND BUDGET JUSTIFICATION 51

16. LITERATURE REFERENCES 51

ANNEXES 54

ANNEX 1: BUDGET AND BUDGET JUSTIFICATIONS 54

ANNEX 2: ROLES AND CREDENTIALS OF RESEARCHERS 57

LIST OF ABBREVIATIONS

AE	Adverse Event
AGYW	Adolescent Girls and Young Women
ART	Antiretroviral therapy
CFR	Code of Federal Regulations (USA)
CTC	Care and Treatment Clinic
DSM	Dar es Salaam Municipality
DSMB	Data Safety and Monitoring Board
FDA	Food and Drug Administration (USA)
GCLP	Good Clinical Laboratory Practice
GCP	Good Clinical Practice
HBsAg	Hepatitis B surface Antigen
HIV	Human Immunodeficiency Virus
HTC	HIV Testing and Counselling
ICC	Intracluster-Correlation Coefficient
ICH	International Conference on Harmonisation
IRB	Institutional Review Board
ITT	Intention To Treat
LTFU	Loss To Follow Up
MDH	Management and Development for Health
MITT	Modified Intention To Treat
MUHAS	Muhimbili University of Health and Allied Sciences
MOP	Manual of Procedures
NACP	National AIDS Control Program
NIMR	National Institute for Medical Research (Tanzania)
OHRP	Office for Human Research Protection
PEPFAR	President's Emergency Plan For AIDS Relief
PI	Principal Investigator
PHDP	Positive Health Dignity Prevention
PrEP	Pre-Exposure Prophylaxis
QC	Quality Control
SAE	Serious Adverse Event
SAP	Statistical Analysis Plan
SMS	Short Message System
STI	Sexually Transmitted Infections
TDF	Tenofovir Disoproxil Fumarate
UP	Unanticipated Problem
USAID	United States Agency for International Development
WHO	World Health Organization

1. PROTOCOL ABSTRACT

**Title:**	Demonstrating service delivery models for effective initiation and retention on PrEP among Barmaids in Dar es Salaam
**Introduction:**	In this double-randomized intervention study, participants will be provided with emtricitabine/tenofovir disoproxil as PrEP. Eligible participants will be randomized first to receive PrEP at their bar workplace or at a government clinic, and second to receive or not receive PrEP Champions intervention (physical and virtual support from an experienced PrEP user) to improve PrEP adherence.. We expect to screen 1204 barmaids in order to have at least 160 who are HIV negative and who agree to enroll in the study. After initial enrollment, follow-up visits will be scheduled every months. HIV testing will be performed at baseline, month 1, 4 and 6; TDF testing at months 2 and 6.
**Objectives:**	Primary objective 1: To assess whether workplace-based PrEP initiation for DSM barmaids at their bar of work increases the rate of PrEP initiation relative to initiation at healthcare facilities with the assistance of a peer navigator (standard of care).<br><br>Primary objective 2: To determine whether workplace-based initiation and provision of PrEP refills to barmaids in DSM at their bar of work increases PrEP retention and adherence relative to routine facility-based PrEP initiation and refilling at healthcare facilities.<br><br>Primary objective 3: To explore preferences for, willingness, and factors influencing uptake of Long-Acting injectable HIV PrEP among female barmaids in Ubungo Municipality**.**<br><br>Secondary objective 1: To assess whether receiving support by an omni-channel (physical and virtual) PrEP Champion, improves PrEP adherence relative to the national standard of care.<br><br>Secondary objective 2: To document the performance of a novel point of care urine assay as a measure of adherence to PrEP.
**Endpoint:**	Primary endpoint 1: Proportion of those offered PrEP who are directly observed to initiate PrEP<br><br>Primary endpoint 2: Adherence to PrEP measured by drug level at 6 months post-enrollment<br><br>Primary endpoint 3: Thesis; Barmaids' preferences, willingness to use, factors influencing uptake of long-acting injectable HIV PrEP and feasibility of implementing injectable PrEP.<br><br>Secondary endpoint 1: Retention in PrEP care measured by refill rates at each study visit<br><br>Secondary endpoint 2: Performance of the urine assay for PrEP adherence will be documented qualitatively. Additionally, onsite test interpretation by health providers will be compared with the test's developers.
**Population:**	1204 women working in bars in Dar es Salaam, Tanzania aged over 15 years

**Phase:**	N/A
**Number of Sites enrolling participants:**	One
**Description of Study Agent:**	The study will involve the provision of emtricitabine 200 mg and tenofovir disoproxil fumarate 300 mg, to be taken orally as a pill once per day for six months. **This use is approved for use by the United States Food and Drug Administration (FDA) and recommended for use to prevent HIV infection by the WHO.**
**Study Duration:**	Twelve months
**Participant Duration:**	**Six months**

2. INTRODUCTION: BACKGROUND INFORMATION AND LITERATURE REVIEW

2.1 BACKGROUND INFORMATION

In Tanzania, there are 1.4 million people currently living with HIV, with a national prevalence of 4.7%. While the country has made considerable strides in addressing the epidemic since the first cases were reported in the 1980s, Adolescent Girls and Young Women (AGYW) continue to be disproportionately affected and vulnerable to HIV. In fact, HIV prevalence triples from 1.3% among adolescent girls aged 15-19, to 4.4% among young women aged 20-24 [1]. This age group bares the greatest HIV burden in the country. Despite being substantially more impacted by HIV, AGYW are often reluctant to seek sexual and reproductive health services due to past experiences of poor quality of care and stigma and discrimination from health care workers [2]. Therefore, more innovative, female-centered, and youth-friendly HIV prevention approaches are necessary.

In 1990, Stein [3] proposed user-dependent HIV prevention methods, specifically ones initiated and managed by women. Oral Pre-Exposure Prophylaxis (PrEP) – one such prevention method – involves the administration of a single antiretroviral (ARV) agent, or dual agents, to HIV-negative individuals prophylactically; to protect them from infection should they be exposed to an infective source. Following the first study of PrEP efficacy, among men who have sex with men (MSM) and transgendered women (TGW) reported in 2010, the concept of a daily pill (emtricitabine (FTC) and tenofovir (TDF), marketed as Truvada to prevent HIV infection has become increasingly accepted as a useful additional biomedical intervention offered to individuals in conjunction with regular testing, counseling, condoms and safe lubricants [4].

The WHO recommended the use of PrEP in 2015 as part of a comprehensive HIV prevention package – including HIV testing, voluntary medical male circumcision (VMMC), and harm reduction interventions for people who use injection drugs – for those at substantial risk for HIV acquisition. Results from trials have shown PrEP as highly efficacious, with a 92% protective effect when used as prescribed [5–9]. These same trials have also demonstrated adherence as key to efficacy, with more adherence to the pill conferring greater protection. Therefore adherence to daily oral PrEP is crucial, for the design and implementation of effective service delivery models for PrEP.

There are several ongoing and planned PrEP demonstration projects in Africa [10,11]. Of biomedical HIV prevention strategies that have been tested, daily oral PrEP has the greatest evidence for protection [12], although adherence was low in two placebo-controlled trials in young African women [13,14]. In Tanzania the use of PrEP was launched in 2018 as part of Tanzania's fourth Health Sector HIV and AIDS Strategic Plan (HSHSP IV 2017-2022), following suit of some other Sub-Saharan countries such as South Africa and Kenya. In the time following PrEP's introduction, the government has initiated demonstration studies to investigate the feasibility of implementing PrEP in the country. The National AIDS Control Program (NACP), through support from ICAP Columbia University USA, Jhpiego (implementing the *sauti* (voices) PrEP program), and HJF medical research international, has enrolled PrEP clients in 9 regions. The country intends to scale up PrEP availability to all 26 regions starting in April 2019, with focus on HIV key populations. Current estimates of PrEP users in Tanzania are between 7800-8300. Gilead's Truvada (TDF/FTC) has been registered and approved in Tanzania, while generic versions of TDF/FTC for prevention is under review (*https://www.prepwatch.org/country/tanzania/*).

PrEP requires a prescription, specialized counseling, regular HIV testing, and close clinical monitoring for side effects and seroconversion through frequent follow-up [15]. However, little evidence exists on the knowledge, attitudes, and perception of female barmaids toward PrEP in sub-Saharan Africa and nothing is known about barmaids' perceptions toward PrEP in Tanzania. Furthermore, because PrEP is still a relatively new biomedical intervention, it is necessary to understand the issues surrounding its implementation into health care systems and

people's perception to this implementation. Regular HIV testing at the time of PrEP initiation and then on an on-going basis, is required for the intervention.

2.2 Problem Statement

Given high efficacy and effectiveness in other populations [16], demonstration projects of open-label PrEP in key populations – the highest risk groups – in Africa are needed to determine feasibility and acceptability of PrEP uptake and adherence. Key questions include: (i) whether those at substantial risk are motivated and able to initiate and sufficiently adhere to oral PrEP to achieve HIV prevention benefits; (ii) which delivery models are most effective; and (iii) how best to generate demand for PrEP in key populations without further stigmatizing them or PrEP use itself. The availability of information about PrEP across Africa has not been formally mapped but is likely suboptimal, both among general and key populations and among healthcare providers; ; and (iv) what their preferences are with regard to long-acting PrEP; if they are willing to use should it be made available and factors that may contribute to their uptake of long-acting injectable PrEP.

2.3 Rationale

In a recent pilot study we conducted interviews and provided HIV testing and counselling (HTC) to 66 barmaids at 7 active bars in Kinondoni district, Dar es Salaam municipality (DSM). Only 4 of the 56 barmaids who completed HTC tested HIV positive (7.1%). Thirty-five percent of interviewed barmaids reported that they engage in sex for money with bar clients – although 80% reported that at least some barmaids in their bar engage in such behavior. In addition, 58% of respondents reported having had multiple sexual partners in the past year, over and above any relations with bar patrons. When asked, 54% of these barmaids stated they were either "very interested" or "somewhat interested" in taking a daily pill to protect against HIV; 61% of those who reported having sex with a bar client for money expressed this interest.

Based on these findings, we believe there is an urgent need to assess the feasibility of PrEP delivery for this population who have low HIV prevalence but are at high risk of HIV infection. In deciding who should be offered PrEP, needs and benefits (HIV prevention) should be balanced with harm (possible adverse events), costs, and feasibility. People who are at

substantial risk of acquiring HIV would achieve the greatest benefit from being able to access PrEP as an additional prevention choice. This study will also provide information on female barmaids 'preferences long-acting injectable PrEP, if they are willing to use and factors both need and predisposing factors that need to be considered by stakeholders in implementation of long-acting injectable PrEP which is already in use in other countries**.**

2.4 Potential Risks and Benefits

2.4.1 KNOWN POTENTIAL RISKS

One risk for emtricitabine/tenofovir disoproxil taken as PrEP is that if someone is HIV-positive they may be at increased risk of developing resistance to the two active drugs. To avoid this we will test individuals for HIV at baseline, month 2 and month 4 during the trial. We will also conduct a detailed assessment of possible acute viral infection based on clinical symptoms; where such symptoms are present initiation will be delayed by one month and a second HIV test performed. Possible adverse reactions to emtricitabine/tenofovir disoproxil noted in its package insert include lactic acidosis/severe hepatomegaly with steatosis, exacerbation of Hepatitis B infection, onset or worsening of renal impairment and loss of bone mineral density. Following the WHO PrEP guidelines [17] Hepatitis B surface antigens will be clinically tested at baseline, with only individuals testing negative considered eligible for the trial. Individuals testing positive will be linked to specialized clinics for further testing and assessed for treatment. Serum creatinine levels will also be assessed at baseline to identify pre-existing renal disease, and at endline (6months). Only individuals with serum creatinine levels less than 60ml/min will be considered eligible to enroll in the trial. A range of other adverse reactions have been seen in clinical trials of emtricitabine/tenofovir disoproxil, including most commonly diarrhea, nausea, fatigue, headache, dizziness, depression, insomnia, abnormal dreams, and rash.

All of these risks are offset by the potential protection emtricitabine/tenofovir disoproxil provides against HIV acquisition in this very high-risk population of young women, who self-report very high levels of sexual activity with multiple partners, including a substantial minority reporting sex in exchange for money or goods. In Tanzania some health care providers from some health facilities have been trained on how to offer PrEP to customers.

Therefore, in this study some selected facilities with trained health care providers on how to offer PrEP in Tanzania will be selected to participate in this study.. Therefore, we will identify four facilities which provide PrEP and select some trained health care providers on PrEP to participate in this study.

2.4.2 KNOWN POTENTIAL BENEFITS

PrEP has been shown to be effective for both men and women. There are studies which involved more than 17 000 people and have demonstrated an overall reduction in HIV acquisition risk of 51% (women relative risk (RR) 0.57; 95% confidence interval (CI) 0.34 - 0.94, and men RR 0.38; 95% CI 0.2 - 0.6). Three studies in which there was high adherence to the study product (>70% of drug detection) showed that PrEP was most efficacious and also that HIV infection was significantly reduced in those studies in which drug detection levels were moderate (41 - 70% detection) [18–20]. In the iPrEx study, a multinational study of HIV-seronegative men or transgender women who have sex with men and with evidence of high risk behavior for HIV-1 infection, Truvada was associated with a 42% reduction in HIV incidence [4,5]. In a trial of serodiscordant heterosexual couples in Kenya and Uganda (Partners PrEP) Truvada was associated with a 75% reduction in incidence of HIV in the seronegative partner [5].

3. STUDY OBJECTIVES

Primary objective 1: To assess whether workplace-based PrEP initiation for DSM barmaids at their bar of work increases the rate of PrEP initiation relative to initiation at healthcare facilities with the assistance of a peer navigator. This will be a person who is trained on social work and counseling (standard of care). Under existing Tanzanian national guidelines, workplace-based HIV testing is standard practice for those in high-risk populations, such as barmaids who often act as informal sex workers. Whether testing positive or negative for HIV, linkage to healthcare is also standard of care. However, self-linkage to HIV care after HIV diagnosis through community testing can be difficult, leading to loss to follow up. Previously, without support from a peer navigator, ART linkage rates in Dar es Salaam public-sector implementation projects have been as low as 30%. Standard of care in this trial will involve active linkage using peer navigators, however we propose to test whether immediate, on-site PrEP initiation leads to

higher rates of initiation compared to facility-based initiation following peer navigator-led active linkage.

Primary objective 2: To determine whether workplace-based initiation and provision of PrEP refills to barmaids in DSM at their bar of work increases PrEP retention and adherence relative to routine facility-based PrEP initiation and refilling at healthcare facilities. Under existing Tanzanian national guidelines, individuals must attend healthcare facilities to obtain refills of ART for treatment. This involves travel cost and waiting time at the clinic, and the potential for stigmatization by attending an HIV CTC. We therefore propose to determine whether having PrEP follow-ups conducted at barmaids' workplace (removing both travel and waiting) will lead to increased retention rates and improved adherence. To avoid possible social desirability bias, we will use urine samples for TDF testing as our primary retention and adherence outcome, since asking women to report their non-use of study HIV drugs has proven problematic in the past [21].

Primary objective 3: To assess barmaids' preferences, willingness to use, feasibility and factors for uptake of long-acting injectable HIV PrEP in Ubungo municipality, in-depth interviews will be conducted with barmaids who have used PrEP for about six months and those who have not been involved with PrEP intervention to learn about their preferences for delivery of and willingness to use long-acting injectable PrEP and factors that contribute to uptake of long-acting injectable PrEP. Also, Key informant interviews will be conducted with different stakeholders working in HIV interventions to understand their views on the feasibility of implementing long-acting injectable PrEP among barmaids, challenges in the implementation of PrEP pills programs and their preferences for service provision and factors that contribute to uptake of long-acting injectable PrEP.

Secondary objective 1: To determine whether being supported by an omni-channel (physical and virtual) PrEP Champion, who provide support and information to clients, improves adherence and retention. Depending on feasibility (owning a smartphone) and preferences of the participants, they will receive physical and/or virtual support from the responsible PrEP Champion who will regularly share standardized informative media with the participants to improve their PrEP knowledge and provide adherence support. We propose to evaluate whether the program omni-channel PrEP Champions is an effective and feasible method to improve

adherence in a high-risk population. We will use self-reported adherence as our effectiveness outcome for this objective, to capture drug use across the whole study period, rather than the shorter window captured by urine-based testing. We will use in-depth interviews with respondents, and key informant interviews with the PrEP Champions, to evaluate the feasibility of omni-channel PrEP Champions, including with respect to rolling it out more widely.

Secondary objective 2: To document the performance of a novel point of care urine assay as a measure of adherence to PrEP. Current tests for adherence to teneforvir – an active agent in PrEP- are predominantly blood (plasma/antibodies) or hair based [22,23]. These traditional methods are invasive and require specialized personnel, equipment and laboratory facilities [22,23]. The University of California San Francisco (UCSF), in collaboration with Alere Rapid Diagnostics have developed a low-cost rapid urine assay that does not require specialized equipment or training. This urine-based assay has been shown to be sensitive (96%), specific (100%) and precise (<15% coefficient of variation) in previous studies [22,23]. The present trial will utilize this point-of-care urine assay and document its performance in a real-world setting.

4. METHODOLOGY

4.1 Description of the Study Design

This study will be an implementation trial to determine the relative effectiveness of two methods for delivering PrEP to barmaids in Dar es Salaam, a population group at high risk for HIV acquisition. The drug provided will be Tenofovir disoproxil fumarate 300 mg and emtricitabine 200 mg, prescribed to be taken orally as a pill once per day. This will be a single-site study and will be collaboratively be conducted by Heidelberg University, the Muhimbili University of Allied Health Sciences (MUHAS) Management and Development for Health (MDH). Implementation of this study will be conducted by MUHAS and MDH, with no clinical care or oversight roles for Heidelberg personnel. Heidelberg personnel will have access to de-identified data only for evaluation purposes, and no contact with human subjects.

Tanzanian research team will visit barmaids in the Ubungo municipal council of Dar es Salaam (formerly part of Kinondoni district, where the piloting was done), and invite them to participate in HIV testing and counseling (HTC). Those who agree to participate will receive

standard HTC procedures as laid out on in the current national Tanzanian guidelines [24]. Standard HTC includes pre-test counselling, a finger-prick based, a rapid HIV test (followed by a second confirmatory test with a second test type should the first test prove positive), and then a post-test counselling. Individuals who test positive for HIV will be linked to HIV care and treatment services at a local HIV Care and Treatment Clinic (CTC), using a routine linkage process from community to facility. At the CTC, individuals will get a confirmatory HIV test and will then be offered immediate antiretroviral therapy (ART) based on current national guidelines for the management of HIV and AIDS [24]. All individuals who test negative for HIV will be actively linked to a PrEP intervention as per the Tanzania National PrEP Framework [25].

The study will involve two serial randomizations. The first randomization will occur at the bar level where barmaids working in a given bar will be offered the ability to initiate PrEP either: (1) at the bar in which the barmaid is working ("workplace-based"), and where they will be recruited to the study by Community based HIV care providers; or (2) at a selected public healthcare clinic already have trained health care workers trained on PrEP and already providing PrEP, as well as providing HIV treatment and care services ("facility-based"). The second randomization will be at the individual level where enrolled PrEP clients will be randomized to either: (A) being supported by an omni-channel PrEP Champion or (B) not being supported by an omni-channel PrEP Champion.

We will identify four facilities to participate in this PrEP provision trial. The criteria for selection will include: (i) existence of HIV care and treatment services; (ii) having a well-established ARV supply chain; (iii) having trained health care providers on how to offer PrEP; and (iv) provide HIV prevention services including condom distribution and comprehensive Positive Health Dignity Prevention (PHDP) services [24]. At each selected facility, we will identify a two well-trained HIV nurse counselors and one ART trained clinician, per WHO recommendations [26] and National PrEP Framework [25]. These staff will be paid for the overtime work they will undertake in supporting this study. The clinicians will be responsible for taking a clinical history and prescribing PrEP. The nurse counsellors will be responsible for providing adherence counselling, issuing PrEP pills and following-up clients for PrEP refills.

Randomization 1: PrEP delivery method

We will randomly assign bars to one of two PrEP delivery groups: facility-based or workplace-based. All women enrolled onto PrEP from each bar will be assigned to their bars' study group, i.e. this is a cluster-randomized design. Bar-level clusters will be used to avoid study contamination and ensure smooth study operationalization.

***<u>Group 1a. Facility-based PrEP delivery</u>*:** All HIV-negative participants in facility-based group bars will be offered the opportunity to initiate PrEP. If they agree to participate, they will be introduced to an assigned peer navigator. The peer navigator will arrange a time to escort the barmaid to their preferred health facility (from the list prepared by the study team) for PrEP services. At the facility, they will be started on emtricitabine/tenofovir disoproxil, with direct observation of the first dose. The study team will actively follow-up the clients to discontinue the participant from PrEP in case they show any signs of side effects. This practice of immediate initiation of treatment followed by discontinuation of treatment among participants with contraindications is comparable to Tanzanian's current guidelines for immediate initiation of ART in HIV-positive patients and PrEP implementation framework.

At the enrolment visit, participants will be scheduled for regular follow-up visits at the facility to monitor medication adherence and safety, once every 2 months, and to receive a PrEP prescription refill on monthly basis.. These visits will include HIV testing, creatinine levels, screening and treatment for STIs as required, and adherence counselling. All these services are standard of care for monitoring patients on treatment and fulfill monitoring requirements in national PrEP and HIV treatment guidelines [24].

***<u>Group 1b. Workplace-based PrEP delivery</u>*:** HIV-negative participants in workplace-based group bars will be offered the opportunity to initiate PrEP at the bar immediately. If they agree to participate, they will be started on emtricitabine/tenofovir disoproxil, with direct observation of the first dose. This initiation will be conducted by study healthcare staff who are allowed to prescribe ART as per Tanzania national policy. Again, should test results subsequently show that participants meet for side effects or other exclusion criteria, the study team will actively follow up to discontinue the participant from PrEP. All subsequent visits will be as in the facility-based group but carried out at the workplace with the assistance of the Community based HIV care providers. The Community based HIV care providers will provide refills; conduct monitoring,

and record inconveniences encountered – since this is an important part of the feasibility assessment for this mode of delivery.

Randomization 2: Omni-channel PrEP Champion

We will randomly assign PrEP study participants into two groups. In the intervention group participants will be provided access to the support by an omni-channel PrEP Champion, to help participants adhere to their PrEP routine. Physical support will be provided through group or individual meetings when they come for PrEP refills whereas; virtual support will be via SMS, calls, WhatsApp groups or individual digital conversations. Additionally, clients will receive the national standard of care support, which includes reminders via SMS and phone calls through the health care provider. The PrEP Champions will regularly share informative standardized media including short, Kiswahili animated videos about PrEP use and adherence, essential PrEP messages, pictures, and gifs about PrEP. The media will be developed collaboratively with the National AIDS Control Program (NACP) staff and pre-tested before they are used in the trial. Participants will also be able to ask questions and discuss with and support each other. The WhatsApp messages and physical meetings will be moderated by the trained PrEP Champions. To share the digital information, the PrEP Champions will receive monthly internet bundles from the study team.

In the control group participants will receive the national standard of care, which includes refill reminders via SMS, phone calls and WhatsApp messages as done in the national standard of care. This randomization will be at the individual level. Those who are willing to participate will sign an additional consent form. Participants will be able to drop out at any time and feedback on the utility of the omni-channel PrEP Champions program will be solicited throughout the study. If a participant does not respond to the standard of care reminders and does not show up at her scheduled appointment, the study team will inform the facility or community PrEP lead about the non-responders for further follow up according to the national guidelines.

Follow up: We will follow up all PrEP participants every two months for a 6 month period after PrEP initiation to effectively assess retention and safety. We will define loss to follow-up (LTFU) as a participant missing two consecutive follow-up visits with no contact. Efforts will be made to contact LTFU participants (independent from the randomization groups) by phone to understand the reasons for missed visits. If participants are contacted and decide not to return, we will ask them to participate in a

brief exit interview to understand the reasons for dropout and to ensure there are no safety concerns. However, for study participants who will want to continue with the study we will use the approach for intention to treat analysis.

4.2 Quantitative Surveys and Qualitative interviews

Quantitative surveys will be conducted throughout the trial. At baseline, a survey will be conducted will all potential participants and will capture demographic, socioeconomic, work and sexual/reproductive health information. At enrollment a survey will be conducted among eligible participants to assess their motivation to accept or decline the use of PrEP and enrollment in the study. At midline and endline study periods, additional surveys will be employed. Among other things, the survey will inquire about changes to social and sexual practices since initiating PrEP, experienced side effects and self-reported PrEP adherence.

Semi-structured in-depth interviews with a subset of barmaids (n=30) enrolled in the larger trial (N=160) will be conducted at baseline (month 0), midline (month 2) and endline (month 6) to contextualize clinically derived adherence results. These interviews will explore, among other things, understandings of adherence, reasons for non-adherence, challenges to adherence, strategies to improve adherence, and recommendations to improve adherence. Additional questions will explore the barmaid's understanding of PrEP, the drug's mechanism of action, if they perceive the drug as being effective against HIV acquisition, and if this perception (positive or negative) influences their adherence to PrEP. Also, barmaids who have been successfully using PrEP pills for six months and those who have not been involved with PrEP intervention will be invited for interviews on their preferences, willingness to use and factors that may influence uptake of long-acting injectable PrEP. Drawing on the barmaid's personal experience and those of their friends, interviews will also explore trends in PrEP use (drug self-regulation), including times of illness, low perceived risk, sharing of PrEP pills with others etc. During the endline survey, we will also collect information from barmaids, PrEP Champions and health care workers perceptions, their personal understanding and experience on the omni-channel PrEP Champions intervention. Key informant interviews will be conducted with stakeholders like NGOs, health care providers, National AIDS Control Program who are working in HIV

interventions to learn their views on preferences, willingness to provide and feasibility for implementation of long-acting injectable PrEP.

To ensure a meaningful sample is included, a purposive sampling design will be employed. Bar maids will be selected to participate in this qualitative portion in a manner that promotes diverse perspectives and experiences. Therefore, bar maids of varying age, length of employment, marital status, wealth, and other factors established during preliminary fieldwork, will be sought. Additionally, the Barmaids's sexual behavior (recorded at baseline) will be taken into consideration for inclusion into the study. Researchers will work to include barmaids who have only one partner and are in a monogamous relationship, and those in concurrent relationships. Health care workers and other key stakeholders working in HIV will be selected using communication from the municipal social welfare officer and District HIV Coordinator who will help to provide information on stakeholders working in HIV.

Based on the specific objectives of the study, we will use different data analysis softwares to answer them. Therefore, quantitative data will be analysed using the following software; SPSS, Stata, R and SAS depending on the nature of the outcome variables. NVivo [Latest available version] and MAXQDA will be used for qualitative data management and analysis.

4.3 Schematic of Study Design

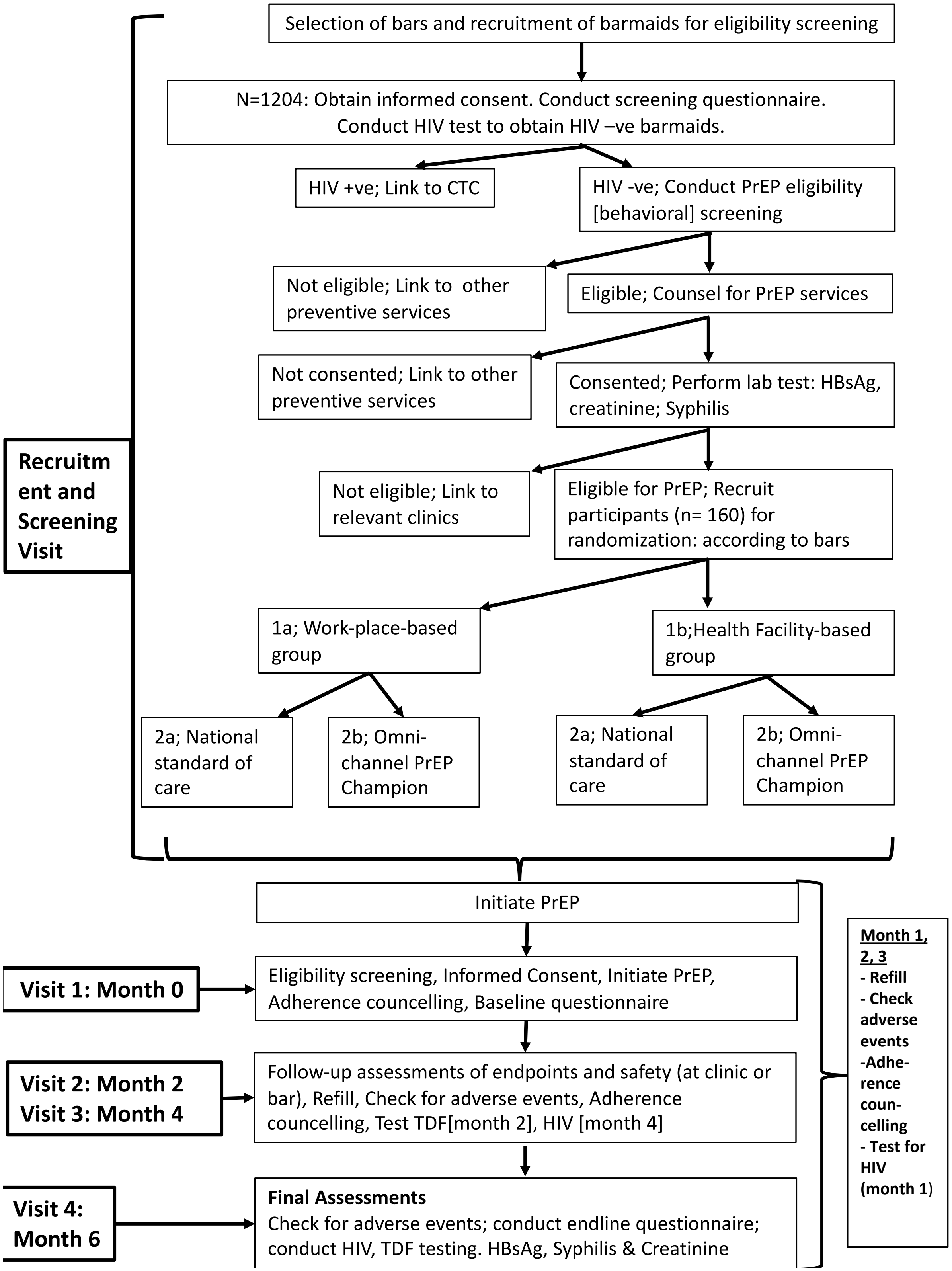

4.3.1 PRIMARY ENDPOINT

Initiation on PrEP measured by the proportion of those offered PrEP who are directly observed to initiate PrEP. The first primary objective of this study is to determine whether offering immediate, on-site PrEP initiation raises initiation rates relative to a peer-navigator led, facility-based initiation process. This endpoint will measure whether the workplace-based initiation process improves uptake, and thus improves the ability of PrEP programs to protect those at high risk of HIV acquisition.

Adherence to PrEP measured by urine drug level at 6 months post-enrollment. The second primary objective of this study is to determine PrEP initiation and drug refill at women's workplaces will improve adherence to PrEP treatment. Measuring adherence to PrEP using urine drug level is the most precise and direct way to measure adherence in the recent past, and thus whether the participant is currently using the drug. Self-report of PrEP use is likely to be biased by social desirability. Ongoing adherence to PrEP is vital to maintaining the protective effect of ART against HIV infection. The primary endpoint will be measured across the two delivery method groups: facility-based and workplace-based PrEP delivery. Women who default from PrEP treatment or are lost to follow up will be assumed to have zero TDF in their urine.

Thesis; this will be a qualitative study that will document Barmaids' preferences, willingness to use, factors influencing uptake of long-acting injectable HIV PrEP and feasibility of implementing injectable PrEP.

4.3.2 SECONDARY ENDPOINTS

Retention in PrEP care measured by refill rates at each study visit; This secondary endpoint will be analyzed in two ways: first, comparing the omni-channel PrEP Champion intervention to no omni-channel PrEP Champion intervention to evaluate secondary objective 1; and second to compare facility- and workplace-based PrEP delivery groups as a supporting analysis for the primary objective. Retention in care reflects the proportion of those initiating care who remain accessible to programs, and thus potentially able to take PrEP.

Documentation of the performance of a point of care urine assay as a measure of adherence to PrEP; will be carried out qualitatively. Health providers will be asked about their experience with the test, including ease of use and interpretation. Additionally, the interpretation of the test onsite will be compared with those conducted by the test's designers as quality control to assess the extent to which they align.

4.3.3 EXPLORATORY ENDPOINTS

1. Level of HIV-related knowledge, including PrEP
2. Characteristics of those accepting and declining to initiate PrEP
3. Proportion of those initiating PrEP retained in care at each study visit
4. Proportion of those initiating PrEP reporting >85% adherence at each study visit
5. Proportion of those initiating PrEP reporting side-effects
6. Number of HIV seroconversions at each study visit
7. Change in number of sexual partners reported in past 30 days from initiation to each study visit
8. Proportion of women reporting consistent condom use at baseline and final visit
9. Proportion of those initiating PrEP with STI symptoms at each study visit
10. Proportion of those initiating PrEP receiving a routine follow-up.
11. Proportion of those initiating PrEP declining being part of the omni-channel PrEP Champions intervention at baseline and at each study visit.
12. Proportion of those initiating PrEP with Tenofovir urine levels compatible with weekly dosing, dosing in the past 2 to 3 days, and dosing within the past 24 hours.

5. STUDY ENROLLMENT AND WITHDRAWAL

5.1 Participant Inclusion Criteria

Individuals must meet all of the following criteria in order to be eligible to participate in this study:

- Identify as female and report being aged 15 years or older

- Working in a licensed bar in Ubungo Municipality Dar es Salaam Municipality(DSM) as a barmaid or server
- Provide signed and dated written informed consent
- State that they are willing to comply with all study procedures and available within DSM for the duration of the study
- Be able to take oral medication and be willing to adhere to the medication regime
- Ability to consent
- A working experience of at least 2 months
- Barmaids who have used PrEP pills consistently for six months and those who have not used PrEP will be recruited for qualitative interviews on long-acting injectable PrEP

5.2 Participant Exclusion Criteria

- Report past diagnosis of renal disease
- Report past diagnosis of diabetes
- Report past diagnosis of hypertension
- Report current use of nephrotoxic medication
- Report flu-like symptoms that may represent acute HIV infection
- Report currently taking any of the following medications:
    - Any medicine containing emtricitabine or tenofovir disoproxil fumarate
    - Any medicine containing tenofovir alafenamide
    - Any medicine containing lamivudine
    - Adefovir
    - Didanosine
    - Atazanavir
    - Ledipasvir with sofobuvir
    - Darunavir
    - Lopanavir with ritonavir
- Report bone pain or other bone problems
- Test positive for HIV or be unwilling to complete HIV counselling and testing.
- Test abnormally low for renal function using a serum creatinine test (<60ml/min).

- Test positive for Hepatitis B based on a hepatitis B surface antigen (HBsAg) test.

5.3 Strategies for Recruitment and Retention

The study aims to recruit 160 HIV negative barmaids to initiate the PrEP intervention. We expect to screen 1204 barmaids in order to recruit these 160 women. There will be only one study site in Dar es Salaam and all individuals will be recruited in Tanzania by locally employed staff.

Participant recruitment will be carried out by visiting licensed bars in DSM, after an initial telephone conversation with the license-holder or representative. On arrival at the bar, the study team will work with the on-duty manager to make a list of all women working as barmaids providing drinks directly to customers. The bar manager will be given a small payment for the time taken to complete the listing and for the inconvenience of the study team's presence. Every woman on this list will then be invited to participate in a screening process including a questionnaire and HIV counselling and testing (HTC). Barmaids will be interviewed in within private tinted mobile vehicles or in a conducive and private place which will be proposed by the barmaids in order to maintain confidentiality. At the initial recruitment stage, potential participants will be offered a small gift (~US$3) to compensate them for the time spent away from their work while completing screening activities. This will be provided at the end of the screening process, unconditional on study inclusion. Approval and support for the study will be obtained from the District Medical Officer of each district in Dar es Salaam where the study will be conducted.

No vulnerable individuals will be targeted for recruitment.

5.4 Participant Withdrawal or termination

5.4.1 Reasons for Withdrawal or Termination

Participants are free to withdraw from participation in the study at any time upon their requests and on their will without being forced or intimidated. The participants will be given clear explanation on the study, process and all procedures that will be done. The issue of withdrawal will be explained clearly that they can stop to participate in the study at any time and that their

withdrawal from the study will have no influence on how they will be treated or handled afterwards.

An investigator may terminate participation in the study (i.e. stop provision of PrEP) if:

- A participant tests positive for HIV
- A participants' renal function drops abnormally low
- Any clinical adverse event (AE), laboratory abnormality, or other medical condition or situation occurs such that continued participation in the study would not be in the best interest of a participant
- A participant ceases to work as a barmaid in Dar es Salaam

Apart from the above reasons for withdrawal or termination of participation in the study, the informed consent has a component that explains about stopping participation and that it will not impose any penalty to the participant.

5.4.2 Handling of Participant Withdrawals or termination

Participants who discontinue PrEP, but otherwise will remain eligible for the study, will remain eligible for follow-up visits at their healthcare facility or bar workplace. Study team members will continue to reach out to these individuals by phone to invite them to scheduled follow-up visits – in order to capture any adverse events or unanticipated problems. Withdrawn or discontinued participants will not be replaced, since such events represent a meaningful endpoint for analysis.

5.5 Premature Termination or Suspension of Study

This study may be temporarily suspended or prematurely terminated if there is sufficient reasonable cause. Written notification, documenting the reason for study suspension or termination, will be provided by the suspending or terminating party to Donna Spiegelman, all funding agencies and all relevant IRBs. If the study is prematurely terminated or suspended, the PI will promptly inform the IRB and will provide the reason(s) for the termination or suspension.

Circumstances that may warrant termination or suspension include, but are not limited to:

- Determination of unexpected, significant, or unacceptable risk to participants

- Insufficient compliance to protocol requirements
- Data that are not sufficiently complete and/or evaluable
- Determination of futility

The study may resume once concerns about safety, protocol compliance, data quality are addressed and satisfy the sponsor, IRB and/or FDA.

6. STUDY AGENT

6.1 Study Agent(s)and Control Description

6.1.1 ACQUISITION

MDH is the HIV implementing partner in Dar es Salaam region and they are also implementing the PrEP program in Ubungo Municipality following the National PrEP Framework. It is in view of this we have agreed to work with MDH in this trial so that we can integrate the trial with the PrEP program in the municipality.

6.1.2 FORMULATION, APPEARANCE, PACKAGING, AND LABELING

Emtricitabine/Tenofovir disoproxil Mylan is available as a light green, film-coated, capsule shaped, biconvex tablet, of dimensions 19.80 mm x 9.00 mm, debossed with 'M' on one side of the tablet and 'ETD' on the other side. It is available either as a bottle or blister pack of 30 film-coated tablets. Emtricitabine/Tenofovir disoproxil Mylan is manufactured by Mylan Inc. and its subsidiary Matrix Laboratories Limited, and has been judged bioequivalent to Truvada. Emtricitabine/Tenofovir disoproxil Mylan is a commercially marketed product, and is available for humans in the form, route and dose planned in this trial.

6.1.3 PRODUCT STORAGE AND STABILITY

Emtricitabine/Tenofovir disoproxil should be stored at 25 degrees Celsius, with excursions permitted to 15-30 degrees Celsius.

6.1.4 PREPARATION

No preparation of Emtricitabine/Tenofovir disoproxil is required by either healthcare provider or participants.

6.1.5 DOSING AND ADMINISTRATION

Emtricitabine/Tenofovir disoproxil will be dosed as a single tablet (containing 200 mg/300 mg of emtricitabine and tenofovir disoproxil fumarate) once daily taken orally with or without food.

6.1.6 ROUTE OF ADMINISTRATION

The route of administration of PrEP will be oral only.

6.1.7 STARTING DOSE AND DOSE ESCALATION SCHEDULE

1 tablet (200 mg/300 mg of emtricitabine and tenofovir disoproxil fumarate). No escalation.

6.1.8 DOSE ADJUSTMENTS/MODIFICATIONS/DELAYS

None

6.1.9 DURATION OF THERAPY

The therapy will last for six months.

6.1.10 TRACKING OF DOSE

Dose/adherence monitoring will be based on self-reported use evaluated every month, as well as pill counts of remaining pills at each visit.

7. STUDY PROCEDURES AND SCHEDULE

7.1 Study Procedures/Evaluations

7.1.1 STUDY SPECIFIC PROCEDURES

- A medical history of each participant will be taken by clinicians at the sites. The history will include medication history including whether they are taking any over-the-counter medications. Based on this the Tanzanian healthcare provider will assess if they are taking any proscribed medications per WHO recommendations.

- A brief physical examination for vital signs, including height, weight and blood pressure will be completed by the clinical team.
- The clinical team will collect a finger-prick blood sample for point-of-care HIV and Hepatitis B surface antigen testing; , TDF testing for PrEP adherence, and will conduct a blood draw for laboratory-based serum creatinine, and syphilis testing.
- The results of all rapid tests will be provided to study participants at the time of testing. Serum creatinine tests will be discussed with participants if the results are abnormal.
- Standard HIV counselling will be provided to participants whether they test positive or negative, per national Tanzanian guidelines.

Adherence to emtricitabine/tenofovir disoproxil will be measured in three ways at each two-monthly visit. First, by self-reported adherence in terms of percentage of doses taken at the agreed time of day on the correct day. Second, by pill count of returned pills at the visit. Third, by a point-of-care urine assay for TDF. Patient-reported outcomes will be ascertained through a short questionnaire at each trial visit, in addition to the collection of baseline data prior to enrolment.

7.1.2 STANDARD OF CARE STUDY PROCEDURES

The trial will be implemented as per the National PrEP framework.

HTC provided at the workplace is standard of care based on national guidelines [24]. Standard HTC includes pre-test counselling, a finger-prick based, a rapid HIV test (followed by a second confirmatory test with a second test type should the first test prove positive), and post-test counselling. Individuals who test positive for HIV will be linked to HIV care and treatment services at a local HIV Care and Treatment Clinic (CTC).

7.2 Laboratory Procedures/Evaluations

All individuals willing to participate in screening will receive rapid HIV testing and counselling according to national guidelines [24]. Blood for Hepatitis B and baseline creatinine testing will be collected and sent to a local accredited laboratory at MUHAS, which will adhere to Good Clinical Laboratory Practice (GCLP) standards. Follow-up testing will occur as shown below.

Visit	Onsite workplace test	Local laboratory test
Recruitment and screening visit	HIV rapid test	Hepatitis B surface antigen test<br>Creatinine test<br>Syphilis test
Month 1	HIV rapid test	
Month 2	TDF urine assay test	
Month 4	HIV rapid test	
Month 6	HIV rapid test<br>TDF urine assay test	Hepatitis B surface antigen test<br>Creatinine test<br>Syphilis test

7.3. Study Schedule

7.3.1 SCREENING

Recruitment and Screening visit

- Recruitment of barmaids for screening and PrEP intervention
- Obtain informed consent of potential participant verified by signature on screening informed consent form.
- Conduct screening questionnaire.
- Obtain demographic information and medical, medication, alcohol and tobacco use history.
- Review medical history to determine eligibility based on inclusion/exclusion criteria.
- Review medications history to determine eligibility based on inclusion/exclusion criteria.
- Perform medical examinations needed to determine eligibility based on inclusion/exclusion criteria. Specifically we will perform:
    - A point-of-care HIV test as part of standard HIV counselling and testing;
    - A point-of-care Hepatitis B surface antigen (HBsAg);and
    - Blood sample collection for a local, laboratory-based test of serum creatinine and syphilis.
- Schedule study visits for participants who are eligible and available for the duration of the study.

- The screening visit will require between 1.5 and 2 hours to complete

7.3.2 ENROLLMENT/BASELINE

Baseline Visit (Visit 0, Month 0)

- Obtain informed consent of potential participant verified by signature on study informed consent form.
- Verify inclusion/exclusion criteria.
- Division into the two study randomization groups 1 and 2
- Directly observe the first dose of emtricitabine/tenofovir disoproxil being taken and provide 28 (1 month) days of emtricitabine/tenofovir disoproxil.
- Counsel participant on adherence to emtricitabine/tenofovir disoproxil and need to contact the study if any changes in health arise.
- Conduct baseline questionnaire

7.3.3 FOLLOW-UP

Refill Visit 1 (Visit 1 Day 30 +/- 7; Month 1)

- Provide additional 28-days (1 month) supply of medication to the participant
- Check on adverse effects
- Adherence Counselling
- Test for HIV

Follow-up Visit 1 (Visit 2, Day 60+/- 7; Month 2)

- Record adverse events as reported by participant or observed by investigator.
- Record vital signs, results of screening for Syphilis or other health conditions.
- Conduct urine tests TDF testing for adherence.
- Provide additional 28-days (1 month) supply of medication to the participant
- Record participant's adherence to treatment program.

- Re-counsel the participant on adherence to emtricitabine/tenofovir disoproxil and need to contact the study if any changes in health arise.
- Visit 2 will require between 1.5 and 2 hours to complete

Refill Visit 2 (Visit 3, Day 90 +/- 7; Month 3)

- Provide additional 28-days (1 month) supply of medication to the participant
- Check on adverse effects
- Adherence Counselling

Follow-up Visit 2 (Visit 4, Day 120+/- 7; Month 4)

- Record adverse events as reported by participant or observed by investigator.
- Record vital signs, or other health conditions.
- Conduct HTC including a rapid HIV test.
- Provide additional 28-day (1-month) supply of medication to the participant
- Record participant's adherence to treatment program.
- Re-counsel the participant on adherence to emtricitabine/tenofovir disoproxil and need to contact the study if any changes in health arise.
- Visit 4 will require between 1.5 and 2 hours to complete

Refill Visit 3 (Visit 5, Day 150 +/- 7; Month 5)

- Provide additional 28-days (1 month) supply of medication to the participant
- Check on adverse effects
- Adherence Counselling

7.3.4 FINAL STUDY VISIT

Final Study Visit (Visit 6, Day 180 +/- 7; Month 6)

- Record adverse events as reported by participant or observed by investigator.
- Record vital signs or other health conditions.
- Collect blood for creatinine and syphilis testing.
- A point-of-care Hepatitis B surface antigen (HBsAg)
- Collect urine for TDF testing for adherence

- Test for HIV
- Record participant's adherence to treatment regimen.
- Provide additional 28-days (1-month) supply of medication to the participant
- Provide final HIV prevention counselling to participant.
- Conduct endline questionnaire
- Inform of continued follow-up support to care by the national program
- Visit 6 will require between 1.5 and 2 hours to complete

7.3.5 EARLY TERMINATION VISIT

At an early termination visit, if the participant is willing, all the above procedures and evaluations conducted for a final study visit will be conducted.

7.3.6 SCHEDULE OF EVENTS TABLE

Procedures	Recruitment and Screening Visit	Baseline Visit 0	Visit 1 (refill)	Follow-up (Visit 2)	Visit 3 (refill)	Follow-up (Visit 4)	Visit 5 (refill)	Final Study Visit (Visit 6)
Selection of bars and recruitment of barmaids for screening	X							
Eligibility	X	X						
Screening/ Baseline/ Endline questionnaire	X	X						X
Informed consent	X	X						
Demographics	X							
Medical history	X							
Randomization 1 (facility- vs. workplace-based)		X						
Syphilis testing	X							X
HBsAg test	X							X
Serum creatinine test	X							X
HIV test	X		X			X		X
Schedule study visits	X							
Provide 28 days of emtricitabine/tenofovir disoproxil		X	X	X	X	X	X	X
Randomization 2 (Omni-channel PrEP Champion)		X						
Record vital signs				X		X		X

Procedures	Recruitment and Screening Visit	Baseline Visit 0	Visit 1 (refill)	Follow-up (Visit 2)	Visit 3 (refill)	Follow-up (Visit 4)	Visit 5 (refill)	Final Study Visit (Visit 6)
TDF in urine				X				X
Counsel on adherence		X	X	X	X	X	X	
Record adherence				X		X		X
Final HIV prevention counseling								X
Information of follow up support by the national program								X
Adverse event evaluation			X	X	X	X	X	X

7.4 Concomitant Medications, Treatments, and Procedures

None.

7.5 Prohibited Medications, Treatments, and Procedures

None.

7.6 Prophylactic Medications, Treatments, and Procedures

Not applicable.

7.7 Rescue Medications, Treatments, and Procedures

Not applicable.

7.8 Participant Access to Study Agent At Study Closure

Since the trial is working in close collaboration with MDH who are implementors of the PrEP national program there will be no problem at the end of the study to link participants to PrEP service provided by the national PrEP program if they wish.

8. ASSESSMENT OF SAFETY

8.1 SPECIFICATION OF SAFETY PARAMETERS

8.1.1 DEFINITION OF ADVERSE EVENTS (AE)

Adverse event means any untoward medical occurrence associated with the use of an intervention in humans, whether or not considered intervention-related (21 CFR 312.32 (a)).

8.1.2 DEFINITION OF SERIOUS ADVERSE EVENTS (SAE)

Serious adverse event or serious suspected adverse reaction. An AE or suspected adverse reaction is considered "serious" if, in the view of either the investigator or sponsor, it results in any of the following outcomes: death, a life-threatening adverse event, inpatient hospitalization or prolongation of existing hospitalization, a persistent or significant incapacity or substantial disruption of the ability to conduct normal life functions, or a congenital anomaly/birth defect. Important medical events that may not result in death, be life-threatening, or require hospitalization may be considered serious when, based upon appropriate medical judgment, they may jeopardize the patient or subject and may require medical or surgical intervention to prevent one of the outcomes listed in this definition (21 CFR 312.32 (a)).

8.1.3 DEFINITION OF UNANTICIPATED PROBLEMS (UP)

This study will use the United States Department of Health and Human Services' Office for Human Research Protection's (OHRP) definition of UP. OHRP considers unanticipated problems involving risks to participants or others to include, in general, any incident, experience, or outcome that meets **all** of the following criteria:

- Unexpected in terms of nature, severity, or frequency given (a) the research procedures that are described in the protocol-related documents, such as the IRB-approved research protocol and informed consent document; and (b) the characteristics of the participant population being studied;
- Related or possibly related to participation in the research ("possibly related" means there is a reasonable possibility that the incident, experience, or outcome may have been caused by the procedures involved in the research); and

- Suggests that the research places participants or others at a greater risk of harm (including physical, psychological, economic, or social harm) than was previously known or recognized.

8.2 CLASSIFICATION OF AN ADVERSE EVENT

8.2.1 SEVERITY OF EVENT

For AEs not included in the protocol defined grading system, the following guidelines will be used to describe severity:

- **Mild** – Events require minimal or no treatment and do not interfere with the participant's daily activities.
- **Moderate** – Events result in a low level of inconvenience or concern with the therapeutic measures. Moderate events may cause some interference with functioning.
- **Severe** – Events interrupt a participant's usual daily activity and may require systemic drug therapy or other treatment. Severe events are usually potentially life-threatening or incapacitating.

8.2.2 RELATIONSHIP TO STUDY AGENT

The clinician's assessment of an AE's relationship to study agent (drug) is part of the documentation process, but it is not a factor in determining what is or is not reported in the study. If there is any doubt as to whether a clinical observation is an AE, the event should be reported. All AEs must have their relationship to study agent assessed. In a clinical trial, the study product must always be suspect. To help assess, the following guidelines are used.

- **Related** – The AE is known to occur with the study agent, there is a reasonable possibility that the study agent caused the AE, or there is a temporal relationship between the study agent and event. Reasonable possibility means that there is evidence to suggest a causal relationship between the study agent and the AE.
- **Not Related** – There is not a reasonable possibility that the administration of the study agent caused the event, there is no temporal relationship between the study agent and event onset, or an alternate etiology has been established.

8.2.3 EXPECTEDNESS

The Tanzanian PI will be responsible for determining whether an AE is expected or unexpected. An AE will be considered unexpected if the nature, severity, or frequency of the event is not consistent with the risk information previously described for the study agent.]

8.3 TIME PERIOD AND FREQUENCY FOR EVENT ASSESSMENT AND FOLLOW-UP

The occurrence of an AE or SAE may come to the attention of study personnel during study visits and interviews of a study participant presenting for medical care, or upon review by a study monitor. All AEs including local and systemic reactions not meeting the criteria for SAEs will be captured on the appropriate RF. Information to be collected includes event description, time of onset, clinician's assessment of severity, relationship to study product (assessed only by those with the training and authority to make a diagnosis), and time of resolution/stabilization of the event. All AEs occurring while on study must be documented appropriately regardless of relationship. All AEs will be followed to adequate resolution.

Any medical condition that is present at the time that the participant is screened will be considered as baseline and not reported as an AE. However, if the study participant's condition deteriorates at any time during the study, it will be recorded as an AE. UPs will be recorded in the data collection system throughout the study.

Changes in the severity of an AE will be documented to allow an assessment of the duration of the event at each level of severity to be performed. AEs characterized as intermittent require documentation of onset and duration of each episode.

The PI will record all reportable events with start dates occurring any time after informed consent is obtained until 7 (for non-serious AEs) or 30 days (for SAEs) after the last day of study participation. At each study visit, the investigator will inquire about the occurrence of AE/SAEs since the last visit. Events will be followed for outcome information until resolution or stabilization.

8.4 REPORTING PROCEDURES

8.4.1 ADVERSE EVENT REPORTING

Any study staff member being made aware of an AE will complete a AE Form and submit to a study PI within 72 hours of site awareness. The study PI will report this AE to the Tanzanian National Institute for Medical Research (NIMR) IRB within 3 business days to the Heidelberg IRB within 5 business days. All AEs will be followed until satisfactory resolution or until the site investigator deems the event to be chronic or the adherence to be stable. Other supporting documentation of the event may be requested by the PIs or the DSMB and should be provided as soon as possible.

8.4.2 SERIOUS ADVERSE EVENT REPORTING

The study clinician will complete a SAE Form within the following timelines:

- All deaths and immediately life-threatening events, whether related or unrelated, will be recorded on the SAE Form and submitted to a study PI within 24 hours of site awareness.
- Other SAEs regardless of relationship to the study will be submitted to a study PI within 72 hours of site awareness.

All SAEs will be followed until satisfactory resolution or until the site investigator deems the event to be chronic or the adherence to be stable. Other supporting documentation of the event may be requested by the PIs or the DSMB and should be provided as soon as possible. The study PIs will be responsible for notifying the FDA and NIMR of any unexpected fatal or life-threatening suspected adverse reaction as soon as possible but in no case later than 7 calendar days after the PI's initial receipt of the information.

8.4.3 UNANTICIPATED PROBLEM REPORTING

Incidents or events that meet the OHRP criteria for UPs require the creation and completion of an UP report form. It is the site investigator’s responsibility to report UPs to their IRB and to the study PI. The UP report will include the following information:

- Protocol identifying information: protocol title and number, PI’s name, and the IRB project number;
- A detailed description of the event, incident, experience, or outcome;
- An explanation of the basis for determining that the event, incident, experience, or outcome represents an UP;

- A description of any changes to the protocol or other corrective actions that have been taken or are proposed in response to the UP.

To satisfy the requirement for prompt reporting, UPs will be reported using the following timeline:

- UPs that are SAEs will be reported to the study PIs within 24 hours of the site becoming aware of the event, to NIMR IRB within 3 business days and to the Heidelberg IRB within 5 business days.
- Any other UP will be reported to the study PIs within 48 hours of the site becoming aware of the event, to NIMR IRB within 3 business days and to the Heidelberg IRB within 5 business days.

8.4.4 EVENTS OF SPECIAL INTEREST

None.

8.5 STUDY HALTING RULES

The study will be halted for a safety review if four (4) SAEs are reported, or two (2) SAEs judged by the Steering Committee to be likely related to study procedures.

8.6 SAFETY OVERSIGHT

Safety oversight will be under the direction of a DSMB composed of individuals with the appropriate expertise, including a Tanzanian clinician, clinicians with experience working on antiretroviral treatment or prevention projects and a biostatistician. The DSMB will meet every three months to assess safety and efficacy data on each arm of the study. The DMSB will operate under the rules of an approved charter that will be written and reviewed at the organizational meeting of the DSMB. At this time, each data element that the DSMB needs to assess will be clearly defined.

9. STATISTICAL CONSIDERATIONS

9.1 STATISTICAL AND ANALYTICAL PLANS

A formal Statistical and Analysis Plan (SAP) will be developed prior to enrollment of any participants in this study.

9.2 STATISTICAL HYPOTHESES

Primary endpoint 1: We hypothesize that the proportion of eligible individuals who agree to initiate PrEP and are observed to take the first tablet on Day 0 will be higher in the workplace-based group than in the facility-based group. The null hypothesis is of no difference between the community- and facility-based groups; this will be a superiority comparison.

Primary endpoint 2: We hypothesize that the urine level of TDF in those enrolled in the workplace-based study group will be significantly higher than that in the facility-based group when measured at the 6-month post-initiation visit. The null hypothesis is of no difference between the community- and facility-based groups.

Secondary endpoint 1a: We hypothesize that the average number of refills made per initiated participant in the workplace-based group across the 6 months of study follow-up (i.e. at visits 2 and 3) will be higher than in the facility-based group. The null hypothesis is of no difference between the community- and facility-based groups; this will be a superiority comparison.

Secondary endpoint 1b: We hypothesize that the adherence per participant in the PrEP Champions intervention group across the 6 months of study follow-up (i.e. at visits 2 to 5) will be higher than that of participants in the national standard of care control group. The null hypothesis is of no difference between the two groups; this will be a superiority comparison.

Secondary endpoint 2: none

9.3 SAMPLE SIZE

The study was powered to detect differences of a clinically significant magnitude with 80% power in: (1) initiation; and (2) adherence to PrEP across the workplace-based and facility-based PrEP provision groups. All analyses were performed in SAS 9.4 using proc power (SAS Institute, Cary NC). We used a two-sided Type-I error of α=0.05 for all calculations.

For initiation, the outcome was the percentage of HIV-negative screened women who are directly observed to initiate PrEP. Our null hypothesis was that there is no difference in initiation rates across study arms, and our alternate hypothesis was that the initiation rates differ from one another. Given our novel intervention and study population, a wide variation in PrEP initiation rates by context, it was difficult to anticipate exact initiation rates. Based on data from our pilot

study showing that 34% of the responding barmaids reported being "very interested" in oral PrEP and a further 20% "somewhat interested", we assumed that PrEP initiation will be approximately 25% in the workplace-based arm and 10% in the facility-based arm. To obtain balance and sufficient power (see next paragraph) for our second, more expensive, primary outcome of PrEP adherence, we would need to randomize 800 participants to the facility-based arm and 320 in the workplace-based arm in a 2:5 ratio. Assuming, based on our pilot data, that 7% of screened women are HIV-negative, we will therefore screen 1204 women. Based upon the test for difference between two binomial proportions and allowing for clustering with 8.25 barmaids per bar and a cluster size coefficient of variation of 0.24 as observed in our pilot study [27], screening 1120 women will provide >80% power to see a 15 percentage point difference in initiation rates over a wide range of PrEP initiation rates and ICCs (and in many cases also for a 10 percentage point difference). This can be seen in the table below, with power >80% shown in shaded cells.

**Initiation rates**			**Intracluster-correlation coefficient**			
Facility-based arm	Difference in workplace-based arm		0	0.02	0.05	0.09
0.1	0.05		0.605	0.542	0.462	0.386
	0.10		0.990	0.978	0.949	0.897
	0.15		1.000	1.000	0.999	0.997
	0.20		1.000	1.000	1.000	1.000
0.2	0.05		0.427	0.378	0.321	0.269
	0.10		0.937	0.901	0.837	0.754
	0.15		0.999	0.998	0.992	0.976
	0.20		1.000	1.000	1.000	0.999
0.3	0.05		0.357	0.317	0.270	0.228
	0.10		0.887	0.839	0.763	0.675
	0.15		0.997	0.993	0.979	0.951
	0.20		1.000	1.000	1.000	0.998
0.4	0.05		0.331	0.294	0.251	0.213
	0.10		0.860	0.809	0.731	0.643
	0.15		0.996	0.989	0.972	0.938

	0.20		1.000	1.000	0.999	0.997
0.5	0.05		0.329	0.293	0.251	0.214
	0.10		0.862	0.812	0.735	0.648
	0.15		0.996	0.990	0.975	0.943
	0.20		1.000	1.000	1.000	0.998

For adherence, our sample size was calculated for a two-sample t-test comparing log-transformed Tenofovir (TDF) levels between the facility-based vs. work-based arms at 6 months following initiation[28]. Participants lost to follow-up before 6 months will be assigned a zero value for TDF since there is no other way to access PrEP in Tanzania at present. Previous research indicated that consistent adherence leads to TDF levels of 80 ng/ml with an interquartile range of 50-120 [29,30]. We therefore assumed that the log-transformed TDF levels were normally distributed with a mean of 4.4 and a standard deviation of 0.6. We accounted for clustering as above. As we show in the table below, to achieve 80% power with balanced enrollment across arms as ensured by design, we will need to enroll 160 women to detect a difference of 25% (or 20 ng/mL) between arms. This difference is meaningful based on a previous study comparing TDF levels among PrEP users who did and did not later acquire HIV[30]. An ICC of 0.02 reflects the ICC for using modern family planning methods (excluding condoms) in our pilot study and also corresponds with previous research on the ICC of biomarkers in Dar es Salaam[31]. However, the number of participants required to achieve 80% power is not sensitive to the ICC.

Mean Tenofovir level (ng/mL)		Intracluster-correlation coefficient			
Workplace-based arm	Facility-based arm	0	0.01	0.02	0.05
80	70	672	724	776	932
80	60	138	150	60	192
80	50	54	60	64	76
80	40	26	30	32	38

9.4 Measures to Minimize Bias

9.4.1 ENROLLMENT/ RANDOMIZATION/ MASKING PROCEDURES

The study will involve a cluster two sequential, independent randomized study. First, bars will be randomized at screening into either workplace-based or facility-based PrEP initiation and follow-up. Second, individuals will be concurrently randomized into either PrEP Campion intervention or no- PrEP Champion intervention for adherence and follow-up support. Anticipating that PrEP initiation will be approximately 10% in the facility-based arm vs. 25% in the workplace-based arm based on previously published studies in the region, we will randomize to either the facility-based arm or the workplace-based arm in a 2:5 ratio in order to obtain balance for ascertaining the second, more expensive outcome, 6-month adherence. If, as hypothesized, PrEP initiation is higher in the community- than the facility-based groups, our first primary outcome is powered to account for this unbalanced design. Under our primary hypothesized difference, in which 10% of those offered facility-based PrEP and 25% of those offered workplace-based PrEP join our study, the four study groups for our second primary outcome and secondary outcomes will look as in the table below:

	Workplace-based PrEP provision	Facility-based PrEP provision
Omni-channel PrEP Champion intervention for PrEP adherence	40	40
National standard of care for PrEP adherence	40	40

Individuals who discontinue the study early will not be replaced; since the primary endpoint is measured as urine level of TDF, they will automatically be assigned the value of zero. This non-replacement will not affect power, since by assigning discontinuing individuals a final outcome value no loss of sample size results.

The study will necessarily not be blinded. From the participants' side they will know which of the four study groups they are in based on the intervention they receive; from the investigators' side they will know who is receiving and not receiving workplace-based PrEP delivery and is part of an Omni-channel PrEP Champion intervention group.

9.4.2 EVALUATION OF SUCCESS OF BLINDING

Not applicable.

9.4.3 BREAKING THE STUDY BLIND/PARTICIPANT CODE

Not applicable.

10. DATA HANDLING AND RECORD KEEPING

10.1 Data Collection and Management Responsibilities

Data collection is the responsibility of the Tanzanian trial staff at the site under the supervision of the site PI. The Tanzanian PI is responsible for ensuring the accuracy, completeness, legibility, and timeliness of the data reported. Data will largely be collected using tablet computers using a digital data collection application, such as CommCare app. This data will be automatically uploaded to the application cloud server will be encrypted and password-protected in accordance to the Level 4 data security and storage regulations. Data will be uploaded nightly to the application server using Wi-Fi. Data collected in this way will include AEs, adverse reactions, clinical test and laboratory data.

10.2 Study Records Retention

All study records will be retained at the Tanzanian research site and at Heidelberg University for 5 years. Paper records (i.e. signed consent forms) will be stored either in a locked cabinet or will be scanned and kept on an encrypted storage device in Tanzania. Electronic records will be maintained on an encrypted storage device. Anonymized data and all anonymized study records at both Heidelberg and MUHAS will be retained for 5 years after study closure.

10.3 Protocol Deviations

A protocol deviation is any noncompliance with the clinical trial protocol, GCP, or MOP requirements. The noncompliance may be either on the part of the participant, the investigator, or the study site staff. As a result of deviations, corrective actions are to be developed by the site and implemented promptly. These practices are consistent with ICH E6:

- 4.5 Compliance with Protocol, sections 4.5.1, 4.5.2, and 4.5.3
- 5.1 Quality Assurance and Quality Control, section 5.1.1
- 5.20 Noncompliance, sections 5.20.1, and 5.20.2.

It is the responsibility of the site to use continuous vigilance to identify and report deviations within 3 working days of identification of the protocol deviation, or within 7 working days of the scheduled protocol-required activity. Protocol deviations must be sent to the local IRB per their guidelines. The Tanzanian site PI and Tanzanian healthcare providers are responsible for knowing and adhering to their IRB requirements.

10.4 QUALITY ASSURANCE AND QUALITY CONTROL

QC procedures will be implemented beginning with the data entry system and data QC checks that will be run on the database will be generated. Any missing data or data anomalies will be communicated to the site(s) for clarification/resolution.

The investigational site (Dar es Salaam) will provide direct access to all trial related sites, source data/documents, and reports for the purpose of monitoring and auditing by all study

sponsors, and inspection by local and regulatory authorities. At no time will Heidelberg study team members have access to identifiable data.

10.5 ANALYSIS DATASETS

The primary dataset for analyses relating to primary endpoint 1 will be an Intention-to-Treat (ITT) analysis dataset, containing all participants who were eligible and thus randomized. The primary dataset for all analyses relating to the primary endpoint 2 and secondary endpoints will be a Modified Intention-to-Treat (MITT) analysis dataset, containing all randomized participants who initiated PrEP. Tanzanians will do the main part of data analyses. There are Tanzanians who will use this data for their PhD studies at Heidelberg University. These Tanzanians will need support on scholarly works from their PhD supervisors at Heidelberg University. Therefore all datasets provided to Heidelberg for analysis will be de-identified.

10.5.1 DESCRIPTION OF STATISTICAL METHODS: GENERAL APPROACH

This study will be a parallel group randomized controlled trial, in which individuals are randomized independently into two interventions: receiving workplace-based PrEP vs. standard of care facility-based provision, and receiving the PrEP champion intervention or not.. All inferential tests will use a type-I error rate of 5% and will be two-tailed.

For univariate descriptive statistics, categorical data will be presented as percentages and continuous data as means and standard deviations (if normally distributed) and medians and interquartile ranges (otherwise).

Covariates for analysis of exploratory endpoints and secondary analysis of primary endpoints will be defined in the later SAP.

10.5.2 ANALYSIS OF THE PRIMARY EFFICACY ENDPOINT(S)

For **primary endpoint 1**, the proportion of eligible individuals who initiate PrEP will be measured based on observation of PrEP initiation at Visit 1. The initiation proportion secondary endpoint will be analysed as such using a two-sample test of proportion. The analysis set for this endpoint will be the ITT dataset including anyone who was screened, found to be eligible for PrEP and was randomized.

For **primary endpoint 2**, TDF level will be measured using urine-based TDF level estimation assay. TDF level will be analysed as a continuous measure in single nanograms per milliliter (ng/ML) and will be a single endpoint measure. The primary endpoint will be assessed using a two-sample t-test of log transformed data where the continuous outcome is TDF level and two exposure categories will be study group (workplace-based or facility-based PrEP). The analysis set for this endpoint will be the MITT dataset including anyone who initiated PrEP based on observed taking of their Day 0 pill.

For the test outcome, we will assume that women who default from treatment or are lost to follow-up will have TDF levels of zero.

10.5.3 ANALYSIS OF THE SECONDARY ENDPOINT(S)

Secondary endpoint 1: The number of refills per initiated participant will be measured as the number of return visits (0-6) at which emtricitabine/tenofovir disoproxil was dispensed to the participant. The endpoint will be assessed twice, in both cases using one-way ANOVA. In the first instance, the likelihood of reporting 0,1,2,3,4,5 or 6 refills will be compared between the facility- and workplace-based groups; in the second instance, the likelihood will be compared between being or not being part of the omni-channel PrEP Champion intervention.

The analysis set for this endpoint will be the MITT dataset including anyone who initiated PrEP based on observed taking of their Day 0 pill.

The secondary analysis will involve no covariates; if preliminary analysis finds covariate imbalance, an ordered logistic regression model adjusting for imbalanced covariates will be run. The list of potential covariates that will be assessed will be specified in the SAP. Results will be presented as odds ratios with a 95% confidence interval.

10.5.4 ADHERENCE AND RETENTION ANALYSES

Adherence and retention form an integral part of the primary and secondary endpoint analyses, and are thus described in Sections 9.4.2 and 9.4.3 above.

10.5.5 BASELINE DESCRIPTIVE STATISTICS

All intervention groups will be compared on baseline characteristics, including demographic, behavioral and laboratory measurements, using proportions (categorical variables), means and standard deviations (continuous, normally distributed variables) and medians and interquartile ranges (continuous, non-normally distributed variables). The list of variables that will be assessed will be specified in the SAP. Inferential tests to detect baseline imbalance will be conducted: two-sample test of proportion (binary variables), χ^2 tests (multi-category variables) and t-tests or Wilcoxon Rank Sum tests (continuous data).

10.5.6 ADDITIONAL SUB-GROUP ANALYSES

We will conduct sub-group analyses on the primary and secondary endpoints based on age **(categories: ages 15-24, 25-34, and ≥35 years).**

10.5.7 TABULATION OF INDIVIDUAL RESPONSE DATA

Individual participant data will not be listed by measure or time point in the final analysis or outputs.

11. ETHICS/PROTECTION OF HUMAN SUBJECTS

11.1 Ethical Standard

The investigator will ensure that this study is conducted in full conformity with Regulations for the Protection of Human Subjects of Research codified in 45 CFR Part 46, 21 CFR Part 50, 21 CFR Part 56, and/or the ICH E6.

In accordance with the Tanzanian government's national PrEP framework, which recommends PrEP use for "vulnerable adolescent girls and young women (AGYW) aged 15-24 years", we will include as potential study participants any barmaids who are aged 15 and above. We do not believe that barmaids aged under 18 should be required to obtain parental consent for legal, ethical and practical reasons. Legally, the national PrEP framework does not require parental consent for initiation among those aged 15-17; this is in alignment with the age of consent for HIV self-testing, which has been 15 since 2019. Ethically, asking for parental consent to a participate in a PrEP trial risks identifying these minors as barmaids – a somewhat stigmatized profession linked to sex work – breaking privacy. Practically, many barmaids do not

live with their families (who are often not in the same city) and as people supporting themselves through work are functionally emancipated. For all these reasons we propose to treat 15-17 year old eligible barmaids as emancipated minors who will receive the same information and informed consent forms as participants 18 years of age or older.

11.2 Declaration of Helsinki

This study will be run in accordance with the principles laid out in the Declaration of Helsinki.

11.3 Institutional Review Board

The protocol, informed consent form(s), recruitment materials, and all participant materials will be submitted to the Heidelberg University IRB, the MUHAS IRB and the Tanzanian National Institute for Medical Research (**NIMR**) for review and approval. Approval of both the protocol and the consent form must be obtained before any participant is enrolled. Any amendment to the protocol will require review and approval by both IRBs before the changes are implemented to the study. All changes to the consent form will be IRB approved; a determination will be made regarding whether previously consented participants need to be re-consented. After getting official documents for permission to conduct the study, the study investigators will seek permission to work in the given area from the responsible medical officers and governments authorities like the Regional and district administrative officers.

11.4 Informed Consent Process

11.4.1 INFORMATION DOCUMENTS PROVIDED TO PARTICIPANTS

Consent forms describing in detail the study agent, study procedures, and risks will be given to the participant and written documentation of informed consent is required prior to starting the intervention. The following consent materials are submitted with this protocol:

- Informed consent form for screening evaluation, interview and HTC
- Informed consent form for study participation

11.4.2 CONSENT PROCEDURES AND DOCUMENTATION

Informed consent is a process that is initiated prior to the individual's agreeing to participate in the study and continues throughout the individual's study participation. Discussion of risks and possible benefits of participation will be provided to the participants and their families. All participants will be informed that PrEP has been shown to be effective in reducing the risk of getting HIV by up to 90% if taken every day as directed. Additionally, participants might benefit from HIV testing during the study period, with immediate referral to HIV treatment services in the event they test positive. Other health-related diagnosis discovered during the study will also receive immediate referral for further investigation and/or treatment, and funds for transportation to reach care will be provided. Furthermore, identified findings from the study will be shared with relevant government institutions, such as the Ministry of Health, and may lead to better provision of HIV and sexual health-related services to women working in bars. In addition to adverse effects associated with the PrEP pill discussed above (page 9, section 2.4.1: known potential risks), participants will also be informed about the time commitments associated with participation in the study.

Consent forms will be IRB-approved. The investigator will explain the research study to the participant and answer any questions that may arise. All participants will receive a verbal explanation in terms suited to their comprehension of the purposes, procedures, and potential risks of the study and of their rights as research participants. Participants will have the opportunity to carefully review the written consent form and ask questions prior to signing.

The participants should have the opportunity to think about the study prior to agreeing to participate. The participant will sign the informed consent document prior to any procedures being done specifically for the study. The consent forms will emphasize that participation in the study is voluntary. The participants may withdraw consent at any time throughout the course of the trial. In the event of a withdrawal, the participant will be asked if they agree for the researchers to analyze the information already obtained from them. If they decline, the collected information will be destroyed. A copy of the informed consent document will be given to the participants for their records if they wish to take it; alternatively they will be provided with a study contact card that does not identify the topic of the study. The rights and welfare of the participants will be protected by emphasizing to them that the quality of their medical care will not be adversely affected if they decline to participate in this study.

11.5 Participant and data Confidentiality

Participant confidentiality is strictly held in trust by the participating investigators and staff. This confidentiality is extended to cover testing of biological samples in addition to the clinical information relating to participants. Therefore, the study protocol, documentation, data, and all other information generated will be held in strict confidence. No information concerning the study or the data will be released to any unauthorized third party without prior written approval of the Site Investigator.

Any authorized representative of the sponsor or representatives of the IRB may inspect all documents and records required to be maintained by the investigator, including but not limited to, medical records (office, clinic, or hospital) and pharmacy records for the participants in this study. The clinical study site will permit access to such records.

The study participant's contact information will be securely stored at each clinical site for internal use during the study. At the end of the study, all records will continue to be kept in a secure location for as long a period as dictated by local IRB and Institutional regulations.

Study participant research data, which is for purposes of statistical analysis and scientific reporting, will be transmitted to and stored at the offices of the Tanzanian partner organization. This will not include the participant's contact or identifying information. Rather, individual participants and their research data will be identified by a unique study identification number. The study data entry and study management systems used by clinical sites and Tanzanian research staff will be secured and password protected. At the end of the study, all study databases will be de-identified and archived in Dar es Salaam and Heidelberg.

All data processing procedures will abide by the Basic Data Protection Ordinance (DSGVO) and the Baden-Württemberg Data Protection Act (LDSG BW).

11.5.1 RESEARCH USE OF STORED HUMAN SAMPLES,SPECIMENS OR DATA

- **Intended Use**: Samples and data collected under this protocol may be used to study factors relating to PrEP initiation, retention and adherence. No genetic testing will be performed.

- **Storage**: No samples will be stored after analysis. Data will be stored using codes assigned by the investigators. Data will be kept in password-protected computers. Only investigators will have access to the data.

11.6 Future Use of Stored Specimens

Not applicable.

12. PUBLICATION AND DATA SHARING POLICY

This study will be registered with ClinicalTrials.gov, a trial registry. Results of the trial will be submitted for publication in peer-reviewed journals.

13. STUDY ADMINISTRATION

13.1 STUDY LEADERSHIP

The Steering Committee will govern the conduct of the study. The Steering Committee will be composed of the study PIs and independent subject matter experts.

14. CONFLICT OF INTEREST POLICY

The independence of this study from any actual or perceived influence, such as by the pharmaceutical industry, is critical. Therefore any actual conflict of interest of persons who have a role in the design, conduct, analysis, publication, or any aspect of this trial will be disclosed and managed. Furthermore, persons who have a perceived conflict of interest will be required to have such conflicts managed in a way that is appropriate to their participation in the trial. The study leadership has established policies and procedures for all study group members to disclose all conflicts of interest and will establish a mechanism for the management of all reported dualities of interest.

15. BUDGET AND BUDGET JUSTIFICATION

See attached excel sheet.

16. LITERATURE REFERENCES

1. Population council. Do adolescents and young women want PrEP? New formative findings from Tanzania. 2017.

2. Geibel S, Hossain SM, Pulerwitz J, Sultana N, Hossain T, Roy S, et al. Stigma Reduction Training Improves Healthcare Provider Attitudes Toward , and Experiences of , Young Marginalized People in Bangladesh. J Adolesc Heal. 2017;60:S35–44.

3. Stein ZA. HIV Prevention: The Need for Methods Women Can Use. Am J Public Health. 1990;80:460–2.

4. Grant RM, Lama JR, Anderson PL, McMahan V, Liu AY, Vargas L, et al. Preexposure Chemoprophylaxis for HIV Prevention in Men Who Have Sex with Men. N Engl J Med [Internet]. Massachusetts Medical Society; 2010;363:2587–99. Available from: https://doi.org/10.1056/NEJMoa1011205

5. Baeten JM, Donnell D, Ndase P, Mugo NR, Campbell JD, Wangisi J, et al. Antiretroviral Prophylaxis for HIV Prevention in Heterosexual Men and Women. N Engl J Med [Internet]. 2012;367:399–410. Available from: http://www.nejm.org/doi/abs/10.1056/NEJMoa1108524

6. Thigpen MC, Kebaabetswe PM, Paxton LA, Smith DK, Rose CE, Segolodi TM, et al. Antiretroviral Preexposure Prophylaxis for Heterosexual HIV Transmission in Botswana. N Engl J Med [Internet]. 2012;367:423–34. Available from: http://www.nejm.org/doi/abs/10.1056/NEJMoa1110711

7. Choopanya K, Martin M, Suntharasamai P, Sangkum U, Mock PA, Leethochawalit M, et al. Articles Antiretroviral prophylaxis for HIV infection in injecting drug users in Bangkok , Thailand (the Bangkok Tenofovir Study): a randomised, double-blind , placebo-controlled phase 3 trial. Lancet. 2013;381:2083–90.

8. Donnell D, Baeten JM, Kiarie J, Thomas K, Stevens W, Cohen CR, et al. Heterosexual HIV-1 transmission after initiation of antiretroviral therapy: a prospective cohort analysis. Lancet. 2010;375:2092–8.

9. Grant RM, Anderson PL, Mcmahan V, Liu A, Amico KR, Mehrotra M, et al. Uptake of pre-exposure prophylaxis, sexual practices , and HIV incidence in men and transgender women who have sex with men: a cohort study. Lancet Infect Dis. 2014;14.

10. Cowan F, Delany-Moretlwe S, Sanders EJ, Mugo NR, Guedou F, Alary M. PrEP implementation research in Africa: what is new? J Int AIDS Soc. 2016;19.

11. AVAC. HIV Prevention Research & Development Database. AVAC. 2016.

12. Van Der Straten A, Van Damme L, Haberer JE, Bangsberg DR. Unraveling the divergent results of pre-exposure prophylaxis trials for HIV prevention. Aids. 2012;26:13–9.

13. Ware NC, Wyatt MA, Haberer JE, Baeten JM, Kintu A, Psaros C, et al. What's Love Got to Do With It? Explaining Adherence to Oral Antiretroviral Pre-exposure Prophylaxis (PrEP) for HIV Serodiscordant Couples. J Acquir Immune Defic Syndr. 2012;59.

14. Corneli AL, Deese J, Wang M, Taylor D, Ahmed K, Micro M, et al. FEM-PrEP: Adherence Patterns and Factors Associated With Adherence to a Daily Oral Study Product for Pre-exposure Prophylaxis. Epidemiol Prev. 2014;66:324–31.

15. Castel AD, Feaster DJ, Tang W, Willis S, Jordan H, Villamizar K, et al. Understanding HIV care provider attitudes regarding intensions to Prescribe PrEP. J Acquir Immune Defic Syndr. 2016;70:520–8.

16. Cáceres CF, Koechlin F, Goicochea P, Sow P, Reilly KRO, Mayer KH, et al. The promises and challenges of pre-exposure prophylaxis as part of the emerging paradigm of combination HIV prevention. J Int AIDS Soc. 2015;18.

17. World Health Organization. WHO IMPLEMENTATION TOOL FOR PRE-EXPOSURE PROPHYLAXIS (PrEP) OF HIV INFECTION: MODULE 1 CLINICAL. Geneva; 2017.

18. World Health Organization. Guideline on When To Start Antiretroviral Therapy and on Pre-Exposure Prophylaxis for HIV [Internet]. World Heal. Organ. 2015. Available from: http://www.who.int/hiv/pub/guidelines/earlyrelease-arv/en/

19. Van Damme L, Corneli A, Ahmed K, Agot K, Lombaard J, Kapiga S, et al. Preexposure Prophylaxis for HIV Infection among African Women. N Engl J Med [Internet]. 2012;367:411–22. Available from: http://www.nejm.org/doi/abs/10.1056/NEJMoa1202614

20. Marrazzo JM, Ramjee G, Richardson BA, Gomez K, Mgodi N, Nair G, et al. Tenofovir-Based Preexposure Prophylaxis for HIV Infection among African Women. N Engl J Med [Internet]. 2015;372:509–18. Available from: http://www.nejm.org/doi/10.1056/NEJMoa1402269

21. Van Der Straten A, Stadler J, Montgomery E, Hartmann M, Magazi B, Mathebula F, et al. Women's experiences with oral and vaginal pre-exposure prophylaxis: The VOICE-C qualitative study in Johannesburg, South Africa. PLoS One. 2014;9.

22. Gandhi M, Bacchetti P, Rodrigues WC, Spinelli M, Koss CA, Drain PK, et al. Development and Validation of an Immunoassay for Tenofovir in Urine as a Real-Time Metric of Antiretroviral Adherence. EClinicalMedicine. 2018;2:22–8.

23. Gandhi M, Bacchetti P, Spinellii MA, Okochi H, Baeten JM, Cressey TR, et al. Validation of a Urine Tenofovir Immunoassay for Adherence Monitoring to PrEP and ART and Establishing the Cut-Off for a Point-of-Care Test. J Acquir Immune Defic Syndr. 2019;81:72–7.

24. The United Republic of Tanzania Ministry of Health and Social Welfare. National guidelines for the managment of HIV and AIDS. Dar es Salaam, Tanzania; 2015.

25. Program NA control. Implementation framework for pre-exposure prophylaxis of HIV in Tanzania mainland. Dar es Salaam, Tanzania; 2021.

26. World Health Organization. Consolidated guidelines on the use of antiretroviral drugs for treating and preventing HIV infection: recommendations for a public health approach. Geneva; 2016.

27. Manatunga AK, Hudgens MG. Sample Size Estimation in Cluster Randomized Studies with Varying Cluster Size. Biomereical J. 2001;43:75–86.

28. O'Brien RGO, Muller KE. Unified Power Analysis for t-Tests through Multivariate

Hypotheses. Edwards L, editor. New York: Marcel Dekker; 1998.

29. Musinguzi N, Muwonge T, Thomas K, Baeten JM, Bangsberg DR, Haberer JE. Does Adherence Change When No One is Looking? Comparing Announced and Unannounced Tenofovir Levels in a PrEP Trial. AIDS Behav. 2016;20:2639–43.

30. Donnell D, Baeten JM, Bumpus NN, Brantley J, Bangsberg DR, Haberer JE, et al. HIV Protective Efficacy and Correlates of Tenofovir Blood Concentrations in a Clinical Trial of PrEP for HIV Prevention. Epidemiol Prev. 2014;66:340–8.

31. Barnhart D, Hertzmark E, Liu E, Mungure E, Muya AN, Spiegelman D. Intra-cluster correlation estimates for HIV-related outcomes from care and treatment clinics in Dar es Salaam, Tanzania. Control Clin Trials. 2016;4:161–9.

ANNEXES

ANNEX 1: BUDGET AND BUDGET JUSTIFICATIONS

Primary funder: University of Heidleberg
Budget currency: Euro

	Description		Units /Qty	Frequency	Months	Unit cost	Total cost	LOE %AGE	Total
A	**SALARIES AND WAGES**								
A.1	Project Country PI	Prof. Rose Mpembeni	1	1	9	8.750	78.750	8%	6.300
A.2	Co-investigator	Doreen Kamori	1	1	9	7.188	64.692	6%	3.882
A.3	Co-investigator	Dr. Idda Mosha	1	1	9	7.188	64.692	5%	3.235
A.4	Study coordinator	TBD	1	1	12	1.000	12.000	100%	12.000
A.5	Lead research assistants	TBD	2	1	9	600	10.800	100%	10.800
A.6	Other research assistants	TBD	3	1	3	600	5.400	100%	5.400
A.7	Accountant	TBD	1	1	9	900	8.100	10%	810
	**Subtotal Personnel**								**42.427**
B	**FRINGE BENEFITS**								
B.1		Once	1	6	1	450	2.700	100,0%	2.700
	**Subtotal Fringe Benefits**								**2.700**
C	**CONSULTANT COSTS**								
C.1	NACP Co-investigator	Monthly	1	1	9	3.000	27.000	5%	1.350
C.2	Overtime allowance for clinic doctors and nurse councelors	Monthly	15	1	9	100	13.500	100%	13.500
C.4	Overtime allowance for Community Outreach Volunteers (COV)	Monthly	24	1	9	30	6.480	100%	6.480
	**Subtotal Consultant Costs**								**21.330**
D	**SUPPLIES**								
D.1	Laptop computer	Once	**1**	**1**	**1**	1.500	1.500	100%	1.500
D.2	Printer	Once	**1**	**1**	**1**	200	200	100%	200
D.3	Tablets	Once	**6**	**1**	**1**	300	1.800	100%	1.800
D.4	Office supplies	Monthly	1	1	1	200	200	100%	200
D.5	Translation	Once	20	1	1	12	240	100%	240
D.6	IRB payment	Once	1	1	1	3.000	3.000	100%	3.000
D.8	Drug delivery bags for nurses for bar-based distribution	Once	8	1	1	50	400	100%	400
D.9	Cost of Reagents	Once	1	1	1	6.000	6.000	100%	6.000
D9	Costs for Lab Tests	Once	1	1	1	17.000	17.000	100%	17.000
	**Subtotal Supplies**								**30.340**
E	**TRAVEL**								
E.1	**Field travel (Vehicle Hire)**	**Montlhy**	1	1	9	2.000	18.000	100%	18.000
	**Subtotal Travel**								**18.000**
F	**OTHER DIRECT COST**								
F.1	Training of interviewers	Once	1	1	1	1.300	1.300	100%	1.300
F.2	PrEP	Once	1	1	1	1.460	1.460	100%	1.460
F.3	Training of COV	Once	1	1	1	1.200	1.200	100%	1.200
F.4	Payment to barmaid to recompense for lost time	Once	1.204	1	1	3	3.612	100%	3.612
F.5	Payments to bar managers for faciliation	Once	130	1	1	10	1.300	100%	1.300
F.6	Stakeholders meetings	Once	1	1	1	2.500	2.500	100%	2.500
F.9	Communication (internet costs)	Monthly	11	1	9	15	1.485	100%	1.485
F.10	Tablet Programmomg and data handling	Once	1	1	1	2.000	2.000	100%	2.000
F.11	Airtime to COV for Refill reminders	Monthly	12	1	9	10	1.080	100%	1.080
F.12	Urine Assays Clearance fees	Once	1	1	1	300	300	100%	300
	**Subtotal Other Direct Cost**								**16.237**
G	**Bar Mapping Exercise**	**Once**							
G.1	Research Assistants half perdiem	daily	2	6	1	30	360	100%	360
	Halfperdiem - Governement filed guides	daily	2	6	1	10	120	100%	120
G.2	Transport for RAs - Hired Bajajs	daily	2	6	1	25	300	100%	300
G.3	Transport for supervisors	daily	1	6	1	50	300	100%	300
G.4	Stationeries & photocopying of tools	Once	1	1	1	25	25	100%	25
	**Subtotal Barmapping Costs**								**1.105**
	**PrEP Champion intervention**								
	**Training of PrEP champions**								
	**Venue**	**Daily**	1	3	1	85	255	100%	255
	Travel and half perdiem for PrEP champions	**Daily**	15	3	1	20	900	100%	900
	**Trainers allowances**	**Daily**	8	3	1	50	1.200		1.200
	Refreshments	**Daily**	24	3	1	10	720		720
	Sub Total Training						3.075		3.075
	**Monthly salary**								
	Monthly Salaries for PrEP champions	**Montly**	15	1	9	120	16.200		16.200
	Total for PrEP champion intervention						19.275		**19.275**
H	**Total Direct Costs**								**151.414**
I	**Institutional Overheads**	**Once**	**1**	**1**	**1**	**0**		**100%**	**22.712,10**
	**Winfrida's PhD qualitative Studies and stipend**	**Once**	**1**	**1**	**1**	**1**	**30.000**	**100%**	**30.000,00**
J	**Total Costs**	**Once**	**1**	**1**	**1**	**1**	**1**	**1**	**204.126,10**

BUDGET JUSTIFICATION

1. Salaries and wages

The budget will be paid to the personnel including the Principal investigator [Prof. Rose Mpembeni] who will monitor data collection, oversee all logistics issues related to the study for 9 months; Two co-investigators Dr. Idda Mosha and Dr. Doreen Kamori will supervise data collection, specimen collection, handling, processing and laboratory analysis for 9 months; **the coordinator who will supervise project activities and reporting to responsible persons;** Lead research assistant and other research assistants who will be involved in participants recruitment and data collection and also the accountant.

2. **Fringe benefits**

   The budget proposed will pay for 5% of health insurance costs for the research assistants and will be valid only during the study period.

3. **Consultant costs**

   The budget for consultant costs will be used to pay NACP-MOHCDGEC HIV care expert. Time compensation allowance for doctors and nurses for 9 months, peer navigators for 3 months and the phlebotomist for a period of 2 months.

4. **Equipment and Others supplies**

   The budget for equipment is for procurement of tablets for data collection, the office supplies, desktop computers, printer for data entry. The cost for the items as per current market prices in Tanzania.

   Also costs for MUHAS and NIMR IRB included under this section are as per respective IRB standard operating procedures.

5. **Laboratory tests and consumables**

   The budget is for materials, supplies that are needed to perform laboratory tests (including specimen collection, sample processing and Rapid tests) and specific laboratory tests needed to address objective of the proposed study. The cost for supplies and reagents is based on current market prices in Tanzania.

6. **Other direct costs**

   The budget for other direct costs will cover **bar mapping,** training of research assistants, doctors and nurses who will be assigned to dispense PrEP, to compensate the bar

managers, barmaids and telecommunication costs in sending remainder to the barmaids. Other direct costs covered by the proposed budget include all expenses such as fuel, transport compensation during the follow-up visits and one stakeholder meeting. **The budget will also cover data collection and stipend for one PhD student under this project.**

7. **Institutional overheads**

   This is a 15% of the total study budget that will be paid to MUHAS as per the institution policy in donor funded research projects.

ANNEX 2: ROLES AND CREDENTIALS OF RESEARCHERS

Name	Role	Contributions
Prof. Till Bärnighausen, MD, ScD<br>Prof. Rose Mpembeni, PhD<br>Prof. Donna Spiegelman, ScD<br>Dr. David Sando, MD, MSc, ScD	Principal investigators	As the principal investigators, Profs. Bärnighausen, Mpembeni, Spiegelman and Sando will provide overall technical, fiscal and administrative oversight of the present research study. This includes the conceptualization and development of the research design/methodology; budget development and management; development of institutional agreements; and enforcement of ethical and institutional polices. Additionally, Prof. Mpembeni will provide supervision and direction on the day-to-day data collection.
Dr. Guy Harling, ScD	Co-investigators	Dr. Harling will contribute to the study design, methodology development and offer technical support in quantitative methods and analyses. He will also contribute to the interpretation and reporting of study results.
Dr. Monica Gandhi, MD, MPH		Dr. Monica Gandhi will contribute towards the coordination of the research study and provide technical input in the design and implementation of the study. She will also contribute to the interpretation study data and reporting

		of the results.
Dr. Doreen, Kamori, PhD		Dr. Kamori will offer her clinical and laboratory expertise and contribute to the overall design, methodology and implementation of the study. Additionally, she will participate in the training of data collectors and data collection activities. She will also contribute to the interpretation and reporting of study results.
Dr. Theodora Mbunda MD, PhD		Dr Mbunda is a Research Scientist overseeing the PrEP program in Ubungo MC. She will therefore work to ensure integration of the Trial and the Community based PrEP program in the study area. She will participate in the trial implementation and ensure that the trial conforms with the national PrEP framework and collection of good quality data. She will also contribute in data analysis and writing of the reports and manuscripts.
Dr. Idda Mosha, PhD		Dr. Mosha will provide technical expertise as it relates to development of qualitative design and methodology. Additionally, she will participate in the training of data collectors and data collection activities. She will also contribute to the interpretation of qualitative data and reporting of the results.
Dr. Dale Barnhart, ScD		Ms. Barnhart will contribute to the study design, methodology development and offer technical support in quantitative methods and analyses. She will also contribute to the interpretation and reporting of study results.
Dr. Joy Chebet, DrPH		Ms. Chebet will contribute towards the coordination of the research study and provide technical input in the design and implementation of the study. She will also contribute to the interpretation study data and reporting of the results.

Ms. Winfrida Onesmo, MSc, BC, BA		Ms. Onesmo will use data collected in this study towards her PhD studies. She will also contribute towards the coordination of the research study, contribute in its design, participate in the data collection, interpretation and reporting of the results.
Ms. Hannah Goymann		Ms. Goymann will contribute towards the coordination of the research study and provide input in the design and implementation of the study. She will also contribute to the interpretation study data and reporting of the results
Prof. Albrecht Jahn, MD, PhD, MSc		Prof Jahn will contribute to the study design, methodology development and offer technical support in quantitative methods and analyses. He will also contribute to the interpretation and reporting of study results.
Dr. Sandra Barteit, MSc, MA		Dr. Barteit will contribute to the study design, methodology development and offer technical support in quantitative methods and analyses. She will also contribute to the interpretation and reporting of study results.
Dr. Judith Kipeleka, MD		Dr. Kipeleka will be responsible for clinical activities including screening, taking of samples and recruitment f study participants.
